# Effect of a digital intervention on mental health symptoms in adults with chronic conditions: A three-arm randomized controlled trial

**DOI:** 10.1371/journal.pmed.1005198

**Published:** 2026-08-20

**Authors:** Emily Johnson, Ashley Hyde, Shaina Corrick, Serena Isley, Gail Wright, Justin Ezekowitz, Yinggan Zheng, Dayna Lee-Baggley, Jude Spiers, John C. Spence, Margaret L. McNeely, Stephanie Thompson, Kathleen Ismond, Sarah Tymchuk, Puneeta Tandon

**Affiliations:** 1 Department of Medicine, University of Alberta, Edmonton, Alberta, Canada; 2 Faculty of Nursing, University of Alberta, Edmonton, Alberta, Canada; 3 Canadian PBC Society, Toronto, Ontario, Canada; 4 Department of Psychology, Saint Mary’s University, Halifax, Nova Scotia, Canada; 5 Faculty of Kinesiology, Sport, and Recreation, University of Alberta, Edmonton, Alberta, Canada; 6 Faculty of Rehabilitation Medicine, University of Alberta, Edmonton, Alberta, Canada; 7 Department of Psychiatry, University of Alberta, Edmonton, Alberta, Canada; Massachusetts General Hospital, UNITED STATES OF AMERICA

## Abstract

**Background:**

Anxiety, depression, and fatigue affect >50% of adults across a range of chronic medical conditions, leading to reductions in quality of life. Digital symptom management interventions may address this burden, but clinical trial evidence across diverse conditions is limited, and the added value of human support remains uncertain. This study aimed to determine whether a multicomponent digital intervention, delivered with or without human support, reduces anxiety and depression compared with usual care at 12 weeks in adults with chronic medical conditions, and whether human-supported delivery outperforms self-directed delivery.

**Methods and findings:**

A three-arm parallel-group open-label randomized controlled trial was conducted from February 2023 to December 2024, with online recruitment and delivery across 13 countries. 825 adults (≥18 years) with self-reported chronic medical conditions and internet access were allocated by computer-generated stratified block randomization (1:1:1) to: (i) waitlist control (*n* = 274); (ii) eMPower, a self-directed digital program integrating video-guided movement, breathwork, and meditation practices, a psychology-based coping skills curriculum, and disease education (*n* = 275); or (iii) eMPower + human support consisting of weekly telephone check-ins (≤15 min) from trained nonclinicians (*n* = 276). The primary outcome was change in Hospital Anxiety and Depression Scale (HADS) total score from baseline to 12 weeks in the eMPower + human support arm compared with the control arm, adjusted for baseline score, chronic condition type, age, and sex. Secondary outcomes included HADS anxiety and depression subscales, fatigue (Modified Fatigue Impact Scale [MFIS]), and health-related quality of life (Short Form-12 [SF-12] mental and physical component scores and EQ-5D-5L index score). Twelve-week assessments were completed by 695 participants (84.2%). Primary outcome data were available for 222 participants in the eMPower + human support arm, 214 in the self-directed eMPower arm, and 259 in the control arm. Analyses followed the intention-to-treat principle. In the prespecified primary comparison, eMPower + human support improved HADS total score by 2.9 points (95% CI [2.0, 3.8]; *p* < 0.001). In prespecified exploratory analyses, the self-directed eMPower arm also significantly improved HADS total score, compared with control (mean difference 2.6 points; 95% CI [1.8, 3.5]; *p* < 0.001). No significant differences were observed between intervention arms for any outcome (all *p* > 0.05). Both intervention arms were associated with improvements across prespecified secondary outcomes compared with control. No intervention-related adverse events were reported in any arm. Key limitations include the use of a waitlist control design, which does not control for nonspecific intervention effects; reliance on self-reported diagnoses for most participants; the 12-week follow-up period; and a predominantly female and highly educated sample, which may limit generalizability. Additional registered process-oriented secondary outcomes and exploratory outcomes will be reported in companion publications.

**Conclusions:**

A multicomponent digital intervention with human support significantly reduced anxiety and depression symptoms compared with usual care in adults with chronic medical conditions. Comparable effects between self-directed and human-supported delivery in exploratory analyses highlight potential for scalable, low-resource implementation. Longer-term follow-up and cost-effectiveness analyses are warranted.

**Trial registration:** ClinicalTrials.gov: NCT05786482.

## Author summary

**Why was this study done?:**

- Anxiety, depression, and fatigue are highly prevalent across chronic medical conditions and are major drivers of disability and reduced quality of life.
- Access to effective symptom management support is limited by mobility barriers, geography, cost, and shortages of trained clinicians, creating an urgent need for scalable, low-resource approaches.
- Digital programs show promise, but most randomized trials are small and single-condition, and it remains unclear whether adding human support meaningfully improves outcomes, an uncertainty that directly affects real-world implementation decisions.

**What did the researchers do and find?:**

- In a fully remote, three-arm randomized controlled trial across 13 countries (*n* = 825), researchers compared a waitlist control, a self-guided multicomponent digital program (movement, breathwork/meditation, coping skills, and disease education), and the same program plus brief weekly support from trained nonclinicians.
- At 12 weeks, the program reduced anxiety and depression symptoms and fatigue, and improved quality of life compared with usual care, with all secondary outcomes remaining significant after multiplicity corrections, demonstrating clinically meaningful benefits in a medically complex cohort.
- Outcomes did not differ between the self-directed and human-supported formats.

**What do these findings mean?:**

- A single, cross-condition digital intervention can improve mental health and fatigue outcomes for adults living with diverse chronic conditions.
- Comparable effects with and without weekly check-ins suggest a “digital first” model may be feasible for broad implementation.
- The study measured outcomes only over 12 weeks, most diagnoses were self-reported, and participants were predominantly women with higher education, so longer-term impact and generalizability to more diverse populations remain uncertain.

## Introduction

Chronic medical conditions affect more than 1 billion people worldwide and are a major contributor to disability, premature mortality, and healthcare utilization [1]. Symptoms of anxiety, depression, and fatigue occur in >50% of patients living with a range of chronic conditions [1,2], resulting in reduced daily functioning, quality of life, and capacity for self-management [1,2].

Digital health interventions offer scalable approaches to symptom management by reducing barriers related to geography, mobility, time [3], and limited healthcare resources [4–7], but important evidence gaps remain. A 2024 systematic review of 56 randomized controlled trials (RCTs) (*n* = 7,691) [8] identified that minimally human-supported digital mind-body interventions, while effective, were often underpowered (median sample <150), had high attrition (>40%), and rarely combined psychological and physical components [8]. Most interventions targeted single conditions, despite evidence that fatigue, anxiety, and depression are common across chronic diseases and share overlapping management principles [8]. In addition, limited evidence exists on whether human support is needed to enhance intervention effectiveness [8–10].

The eMPower trial evaluated a multicomponent digital intervention targeting anxiety, depression, and fatigue in adults with diverse chronic conditions. The primary study question was whether the eMPower intervention delivered with human support would improve symptoms of anxiety and depression, as measured by the Hospital Anxiety and Depression Scale (HADS) total score, compared with usual care at 12 weeks. It was hypothesized that human-supported delivery would improve mental health outcomes compared with control, with secondary benefits for fatigue and quality of life, and would outperform self-directed delivery.

## Methods

### Ethics statement

This study was approved by the University of Alberta Health Research Ethics Board–Health Panel (Pro#00122568). All participants provided written informed consent electronically via Research Electronic Data Capture (REDCap) [11] prior to completing baseline assessments and randomization. As the trial was conducted entirely online from a single coordinating center with no in-person visits or local research sites, the University of Alberta Health Research Ethics Board served as the sole ethics board of record for all participants regardless of country of residence. A supplementary consent addressing General Data Protection Regulation (GDPR) requirements was obtained for participants in applicable jurisdictions. All protocol amendments were approved by the Ethics Board prior to implementation.

### Study design and participants

Recruitment occurred between February 12, 2023 and June 16, 2024. The final participant completed 12-week follow-up on December 1, 2024. The trial was registered on ClinicalTrials.gov (Identifier: NCT05786482, Date of registration: March 9, 2023) [12] and is reported in accordance with the Consolidated Standards of Reporting Trials (CONSORT) (see S1 Checklist) [13]. Trial modifications are documented using the CONSERVE-CONSORT Checklist (S2 Checklist). The trial was registered retrospectively, ~25 days after enrollment of the first participant, due to administrative delays including institutional account setup and entry of required protocol fields on ClinicalTrials.gov. No changes were made to the study design, outcomes, or analysis plan between enrollment of the first participant and trial registration, and registration was completed before any primary outcome data were collected. All data analyses were completed after end-of-study (December 1, 2024), with no interim analyses.

Eligible participants were adults (≥18 years) with a self-reported physician-diagnosed chronic physical condition of at least 3 months’ duration requiring ongoing management [14]. Additional eligibility criteria included English proficiency and internet access. Participants reported a primary physician-diagnosed chronic physical condition at screening and provided current medication lists. For participants residing in Alberta, Canada who provided personal health numbers (39%), diagnoses were verified through provincial electronic medical records. For others, reported diagnoses were reviewed by a study physician using medication lists and available clinical information. The total number of chronic conditions per participant was not collected.

At trial initiation, eligibility was restricted to adults ≥50 years. On May 19, 2023 (after 180 participants were enrolled), the minimum age was lowered to ≥18 years following steering committee review to improve inclusivity and align eligibility with the trial’s cross-condition design. Initial exclusion criteria included living with an uncontrolled psychiatric condition (post-traumatic stress disorder, bipolar disorder, or schizophrenia); near-daily suicidality; and Hospital Anxiety and Depression Scale (HADS)-Depression subscale score >10. Psychiatric conditions were considered uncontrolled if participants reported recent hospitalization, acute crisis, or lack of ongoing treatment. On October 21, 2023 (after 321 participants were enrolled), the HADS-Depression >10 exclusion was removed following consultation with psychiatrists and patient partners to improve equitable access, while retaining exclusion for near-daily suicidality and uncontrolled psychiatric illness. All amendments were approved by the University of Alberta Health Research Ethics Board prior to implementation and are detailed in the published trial protocol [12].

### Procedures

Participants were recruited entirely online through multiple channels: email lists and websites of patient partner organizations (including HeartLife, the Canadian PBC Society, the Canadian Liver Foundation, and the Canadian Digestive Health Foundation), social media platforms, online patient forums, and patient conferences. Clinical centers affiliated with the study team were also informed about the trial and provided with advertisement materials. All channels directed interested individuals to the study recruitment website, where they could access study information, provide electronic informed consent, and complete baseline assessments via REDCap [11]. Recruitment prioritized conditions with existing patient partner collaborations (e.g., heart failure, post-transplant, chronic digestive disease, liver disease), while an ‘other chronic condition’ category permitted enrollment of individuals with additional chronic conditions, supporting the intervention’s cross-condition approach. To support data integrity, research staff manually reviewed names and email addresses prior to orientation to prevent duplicate enrollment and maintained tracking logs documenting orientation attendance and study communication. Automated survey timestamps were monitored, with manual follow-up as needed.

### Randomization and masking

Participants were randomized to one of three groups: control, self-directed eMPower, or eMPower + human support. Randomization was conducted electronically in REDCap [11] using computer-generated permuted blocks of size 6 within eight predefined chronic medical condition strata in a 1:1:1 allocation ratio. The allocation sequence was generated by the study statistician and uploaded to REDCap prior to recruitment. Allocation concealment was maintained until assignment. Due to the nature of the intervention, participant blinding was not feasible.

### Interventions

The eMPower intervention, co-designed with patients [15,16], and informed by the capability, opportunity, motivation-behavior (COM-B) model [17], incorporated 22 behavior change techniques to support adherence (S1 Table) [18]. The program consisted of two components:

1. Expert-led breathwork, meditation and movement (e.g., chair exercise, yoga, and Tai-chi) videos at multiple difficulty levels and lengths (full-length routines 20–35 min, short routines 3–10 min)
2. Psychologist-led coping skills curriculum (e.g., values clarification, pacing strategies) based on principles of Acceptance and Commitment therapy (ACT). This was complemented by disease-specific education videos delivered by clinicians with expertise in each condition area, designed to support understanding and self-management (~5–15-min) (see S8 Table for topics). Participants were assigned educational content based on their primary chronic condition reported at enrollment; no additional tailoring was provided for comorbid conditions

Participants in the eMPower + human support arm received weekly telephone check-ins (≤15 min) with nonclinical personnel who completed pre-study training (a 2-day course) in motivational interviewing. Check-ins followed a semi-structured script adapted from previous trials (S2 File) [16,19]. Both intervention groups received weekly newsletters highlighting program content (S3 File) and had the option to join weekly guided group movement sessions. All intervention content, newsletters, and check-in calls were delivered in English.

Control participants were assigned to a waitlist control condition, in which they continued their usual medical care, received weekly emails containing motivational wellness quotes, and were offered access to the self-directed program after the 12-week study period. No restrictions were placed on concomitant treatments.

### Outcomes

The primary outcome was HADS total score at 12 weeks, analyzed using Analysis of Covariance (ANCOVA) with adjustment for baseline HADS total score, chronic condition type, and age and sex, given their known associations with digital intervention engagement and mental health outcomes [20,21]. Secondary outcomes included HADS anxiety and depression subscales (HADS-A, HADS-D), fatigue measured using the Modified Fatigue Impact Scale (MFIS) [22] and the mental and physical component summary scores of the 12-Item Short Form Survey (SF-12 MCS and PCS) [23]. Health-related quality of life was assessed using EQ-5D-5L index scores calculated with country-specific value sets [24]. The EQ-5D-5L Visual Analog Scale (VAS) score was collected and reported descriptively but was not included among the prespecified secondary endpoints for confirmatory testing [25].

Prespecified exploratory analyses included comparisons of the combined intervention arms versus control and comparisons between the two intervention arms. Participants were encouraged to report intervention-related adverse events to the study team throughout the trial period. Condition-specific quality-of-life measures prespecified in the published protocol were collected where applicable; however, because these instruments apply only to subsets of participants and would require condition-stratified analyses with limited power, they are not presented in this primary cross-condition report [12].

Two additional registered secondary outcomes, the Capability, Opportunity, Motivation, Behavior (COM-B) survey assessing behavioral drivers of engagement and a satisfaction and adherence measure, are process-oriented engagement measures being planned for separate implementation focused publications. Registered exploratory outcomes (demoralization, frailty, sleep disturbance, and healthcare usage) are similarly planned for separate focused analyses.

### Sample size and statistical analysis

The sample size was powered for the single prespecified primary comparison: eMPower + human support versus control on HADS total score at 12 weeks. Sample size was calculated using G*Power (Version 3.1.9.7) [26], as described in the published protocol [12]. Under a three-arm omnibus framework, effect sizes of 0.2 for the self-directed eMPower arm and 0.5 for the eMPower + human support arm were assumed. Under these assumptions, and allowing for 20% attrition, a total sample size of 309 participants (103 per arm) was required to detect the prespecified primary comparison (eMPower + human support versus control) with two-sided α = 0.05 and 80% power. The sample size was not separately powered for secondary outcomes; accordingly, secondary analyses may have limited power to detect smaller but clinically meaningful effects, and nonsignificant findings on individual secondary endpoints should not be interpreted as evidence of no effect.

Analyses followed a prespecified hierarchical testing strategy to control the familywise Type 1 error rate. An omnibus ANCOVA first assessed overall differences across the three randomized arms. If the omnibus test was statistically significant (α = 0.05), the single prespecified primary pairwise comparison (eMPower + human support versus control) was examined at α = 0.05. This hierarchical gatekeeping approach, in which the primary comparison proceeds conditional on a significant omnibus test, controls the Type 1 error rate without requiring further multiplicity adjustment, consistent with CONSORT guidance for multi-arm trials [27]. The Hochberg step-up procedure was applied across the six prespecified secondary outcomes (HADS-Anxiety, HADS-Depression, MFIS total, SF-12 PCS, SF-12 MCS, and EQ-5D-5L index score) for the primary comparison (eMPower + human support versus control) to control the familywise Type 1 error rate [28]. As a more conservative sensitivity analysis, the Bonferroni correction was also applied [29], yielding a uniform adjusted threshold of α = 0.05/6 = 0.0083. Both methods are presented in S7 Table.

All analyses followed the intention-to-treat principle. Baseline demographics and clinical characteristics were summarized using descriptive statistics. Normality assumptions were verified with log transformation as needed. Absolute between-group improvement was quantified as the adjusted mean difference between groups derived from the ANCOVA model, while relative improvement was calculated as adjusted between-group difference divided by the mean value in the control group at 12 weeks. The same approach was applied to secondary and exploratory outcomes. Analyses were conducted using SPSS (Version 24.0) [30] and RStudio (Version 2025.05.0).

ChatGPT-4.0 (OpenAI, 2025) was used during final manuscript preparation to enhance the readability and grammar of selected sections of the text. No AI tools were used in the design, conduct, or analysis of the study, nor in the interpretation of results. All AI-assisted text was reviewed, verified, and edited by the authors to ensure accuracy and consistency with the original intended content. The authors retain full responsibility for the final manuscript.

## Results

### Participant flow and baseline characteristics

**Fig 1:**
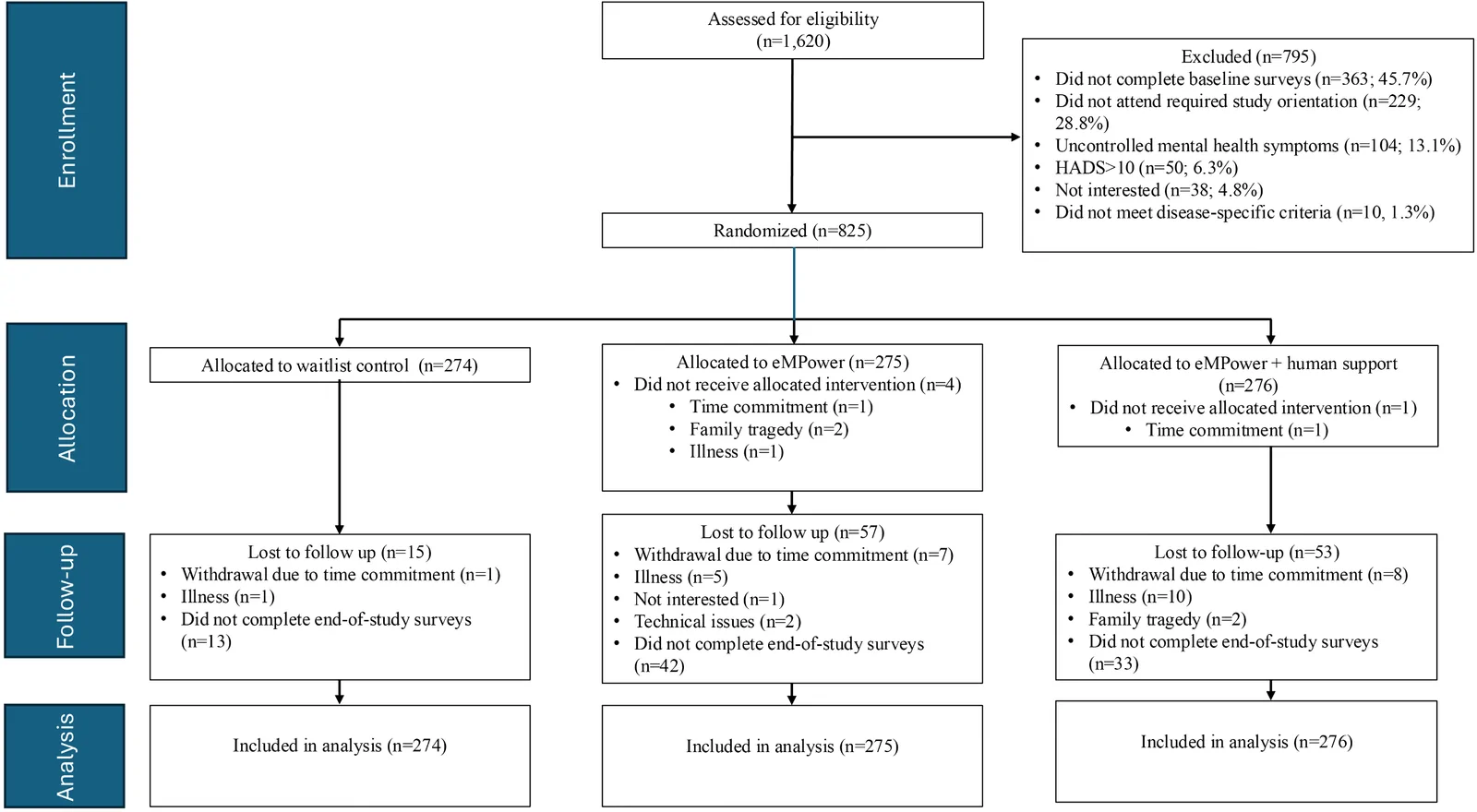
CONSORT flow diagram. Flow diagram showing the number of participants assessed for eligibility, excluded, randomized, allocated to each study arm, lost to follow-up, and included in the intention-to-treat analysis. CONSORT, Consolidated Standards of Reporting Trials.

Of 1,620 individuals who initiated enrollment between February 2023 and June 2024, 825 were randomized (Fig 1). Among the 795 not randomized, primary reasons for exclusion included incomplete baseline surveys (*n* = 363; 45.7%), not attending an orientation with the study team (*n* = 229; 28.8%), and uncontrolled psychiatric symptoms (*n* = 104; 13.1%). Remaining exclusions were due to miscellaneous reasons. Retention was 84.2% (*n* = 695) at 12 weeks. No intervention-related adverse events were reported to the study team throughout the trial period.

Baseline characteristics were balanced across groups (Table 1). Mean ± standard deviation (SD) age was 55.6 ± 12.7 years, with 24% (*n* = 198) over the age of 65. The sample was predominantly female (*n* = 695; 84%) and white (*n* = 730; 88.5%). Participants were enrolled from 13 countries, predominantly Canada (*n* = 675; 81.8%) and the United States (*n* = 120; 14.5%), with remaining participants from the United Kingdom, Australia, Mexico, the Netherlands, Israel, Japan, New Zealand, the Philippines, Portugal, Spain, and Sweden. The most common conditions were Primary Biliary Cholangitis (PBC) (*n* = 184; 22.3%), chronic digestive diseases (*n* = 128; 15.5%), and post-transplant status (*n* = 111; 13.5%). Seventy percent (70%, *n* = 578) had post-secondary education. Participants reported good technology proficiency, with 34% (*n* = 278) reporting moderate and 40% high confidence (*n* = 329) (S2 Table).

**Table 1:** Baseline demographic data and clinical characteristics of randomized participants by treatment arm.

	Total (*n* = 825)	eMPower + human support (*n* = 276)	eMPower (*n* = 275)	Control (*n* = 274)
**Age**				
Mean (SD)	55.6 (12.7)	55.3 (12.3)	56.0 (12.8)	55.6 (13.0)
Median (IQR)	57.0 (47.0, 65.0)	57.0 (48.0, 65.0)	58.0 (46.0, 66.0)	57.0 (47.8, 65.0)
**Sex,** *n* (%)				
Female	695 (84.2%)	229 (83.0%)	234 (85.1%)	232 (84.7%)
**Gender**, *n* (%)				
Woman	684 (82.9%)	224 (81.2%)	229 (83.3%)	231 (84.3%)
**Ethnicity,** *n* (%)				
White	730 (88.5%)	244 (88.4%)	239 (86.9%)	247 (90.1%)
Southeast Asian	19 (2.3%)	6 (2.2%)	8 (2.9%)	5 (1.8%)
Indigenous	17 (2.1%)	6 (2.2%)	8 (2.9%)	3 (1.1%)
South Asian	13 (1.6%)	7 (2.5%)	3 (1.1%)	3 (1.1%)
Black, African, or Caribbean	12 (1.5%)	3 (1.1%)	6 (2.2%)	3 (1.1%)
Latin American	11 (1.3%)	4 (1.4%)	4 (1.5%)	3 (1.1%)
West Asian	2 (0.2%)	1 (0.4%)	1 (0.4%)	—
Arab	1 (0.1%)	1 (0.4%)	—	—
Prefer not to answer	20 (2.4%)	4 (1.4%)	6 (2.2%)	10 (3.6%)
**Marital Status,** *n* (%)				
Married/Living common-law	554 (67.2%)	179 (64.9%)	185 (67.3%)	190 (69.3%)
Single/Never married	124 (15.0%)	46 (16.7%)	41 (14.9%)	37 (13.5%)
Divorced/Separated	120 (14.5%)	45 (16.3%)	38 (13.8%)	37 (13.5%)
Widowed	27 (3.3%)	6 (2.2%)	11 (4.0%)	10 (3.6%)
**Education level, *n* (%)**				
Completed post-secondary school (college/university)	578 (70.1%)	200 (72.5%)	186 (67.6%)	192 (70.1%)
Some college/university	132 (16.0%)	38 (13.8%)	55 (20.0%)	39 (14.2%)
Completed high school	61 (7.4%)	20 (7.2%)	17 (6.2%)	24 (8.8%)
Registered apprenticeship or other trades certificate	38 (4.6%)	15 (5.4%)	12 (4.4%)	11 (4.0%)
No degree, certificate or diploma	16 (1.9%)	3 (1.1%)	5 (1.8%)	8 (2.9%)
**Chronic medical condition,** *n* (%)				
Other chronic medical condition	254 (30.8%)	86 (31.2%)	85 (30.9%)	83 (30.3%)
PBC	184 (22.3%)	61 (22.1%)	62 (22.5%)	61 (22.3%)
Chronic digestive disease(s)	128 (15.5%)	43 (15.6%)	42 (15.3%)	43 (15.7%)
Post-transplant	111 (13.5%)	37 (13.4%)	37 (13.5%)	37 (13.5%)
Cirrhosis	51 (6.2%)	17 (6.2%)	17 (6.2%)	17 (6.2%)
Heart failure	48 (5.8%)	16 (5.8%)	16 (5.8%)	16 (5.8%)
CKD	29 (3.5%)	9 (3.3%)	10 (3.6%)	10 (3.6%)
PSC	20 (2.4%)	7 (2.5%)	6 (2.2%)	7 (2.6%)

Data are presented as mean (SD) and median (IQR) for continuous variables and n (%) for categorical variables. The “Other chronic medical health condition” category included participants with chronic conditions outside the prespecified strata. Sex assigned at birth and self-reported gender identity were collected separately. SD, standard deviation; IQR, interquartile range; PBC, primary biliary cholangitis; PSC, primary sclerosing cholangitis; CKD, chronic kidney disease; — indicates zero participants.

Participants had a substantial baseline symptom burden (Table 2). Mean ± SD HADS total score was 13.5 ± 6.5, with a mean HADS-anxiety score of 8.0 ± 4.0 and a mean HADS-depression score of 5.5 ± 3.5. Fatigue was prominent (MFIS total mean±SD: 45.4 ± 16.0), with participants reporting moderate-to-severe symptoms across physical, cognitive, and psychosocial domains. Quality of life was impaired with SF-12 PCS and MCS scores below population norms (means 39.2 and 42.2, respectively). EQ-5D-5L mean±SD index scores, averaged 0.7 ± 0.2, reflecting moderate impairment.

**Table 2:** Baseline symptom and quality of life scores by treatment arm.

Outcome	Total (*n* = 825)	eMPower + human support (*n* = 276)	eMPower (*n* = 275)	Control (*n* = 274)
**HADS total score, mean** (SD)	13.5 (6.5)	13.5 (6.8)	13.7 (6.2)	13.2 (6.5)
HADS-Anxiety, mean (SD)	8.0 (4.0)	8.0 (4.1)	8.1 (3.8)	7.8 (4.0)
HADS-Depression, mean (SD)	5.5 (3.5)	5.5 (3.6)	5.5 (3.6)	5.4 (3.4)
**MFIS Total,** mean (SD)	45.4 (16.0)	45.8 (17.5)	46.0 (15.0)	44.4 (15.3)
MFIS Physical, mean (SD)	21.8 (7.8)	21.7 (8.2)	22.1 (7.7)	21.5 (7.6)
MFIS Cognitive, mean (SD)	19.3 (8.1)	19.7 (8.9)	19.5 (7.4)	18.7 (7.9)
MFIS Psychosocial, mean (SD)	4.3 (2.0)	4.4 (2.2)	4.4 (2.0)	4.2 (2.0)
**EQ-5D-5L** Index Score, mean (SD)	0.7 (0.2)	0.7 (0.2)	0.7 (0.2)	0.7 (0.2)
EQ-5D-5L VAS, mean (SD)	61.6 (18.8)	60.4 (19.7)	60.8 (18.6)	63.5 (18.1)
**Quality of life (SF-12)**				
SF-12 PCS score, mean (SD)	39.2 (10.6)	39.1 (10.9)	39.0 (10.5)	39.4 (10.6)
SF-12 MCS score, mean (SD)	42.2 (10.3)	41.9 (10.0)	42.0 (10.5)	42.7 (10.4)

Data are presented as mean (SD). Higher HADS and MFIS scores indicate greater symptom burden, while higher SF-12 and EQ-5D-5L scores indicate better health-related quality of life. EQ-5D-5L index scores were calculated using country-specific value sets where available. HADS, Hospital Anxiety and Depression Scale; MFIS, Modified Fatigue Impact Scale; SF-12, Short Form-12; MCS, Mental Component Summary; PCS, Physical Component Summary; VAS, Visual Analog Scale; SD, standard deviation.

### Primary outcome

**Fig 2:**
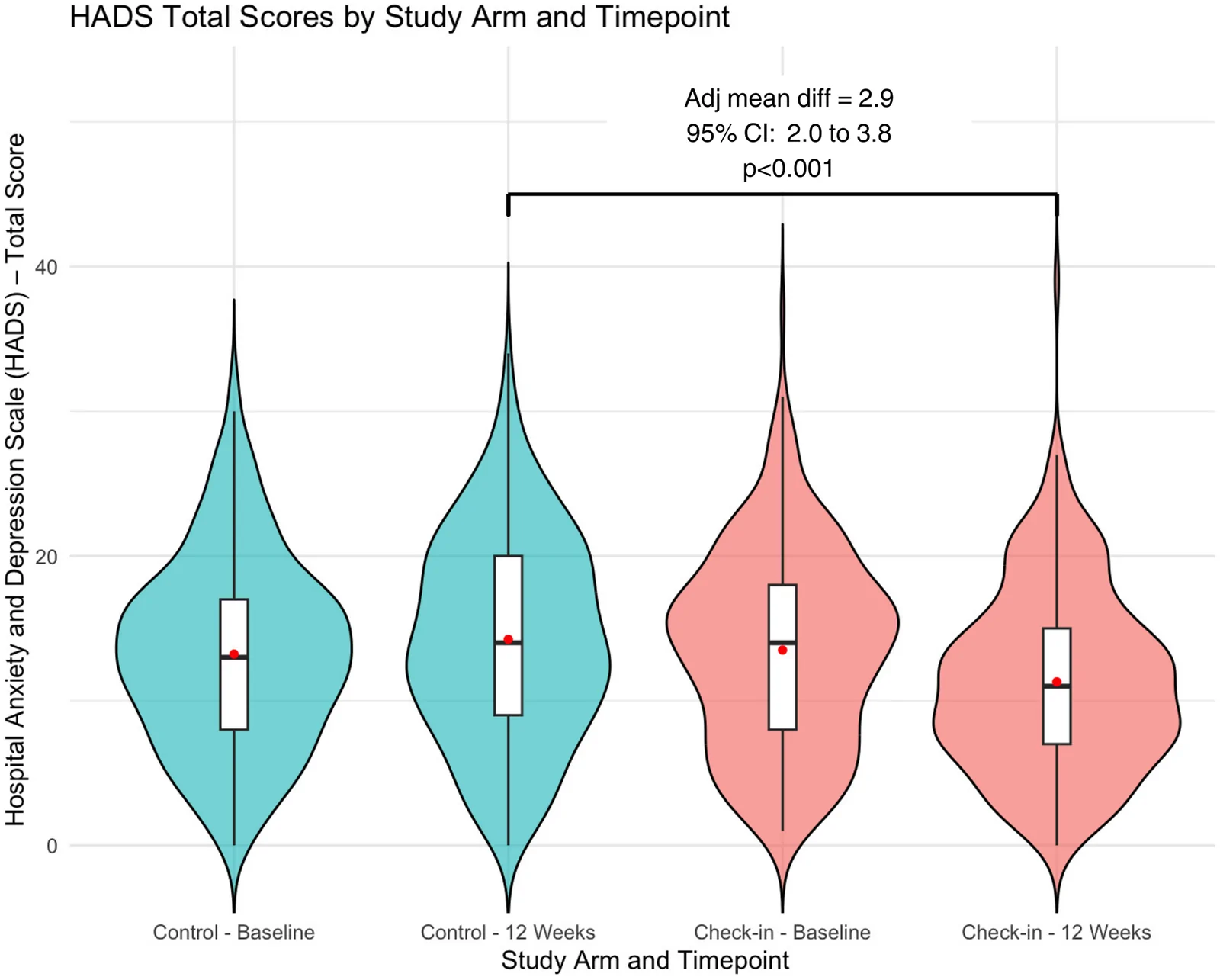
Distribution of psychological distress (HADS total) at baseline and 12 weeks in eMPower + human support vs. control. Violin plots show the distribution of HADS total scores at baseline and 12-week follow-up. Blue represents waitlist control and peach represents eMPower + human support. Higher scores indicate greater psychological distress. Adjusted between-group mean differences with 95% CIs, adjusting for baseline, chronic condition type, age, and sex are reported in Table 3. HADS, Hospital Anxiety and Depression Scale; ANCOVA, Analysis of Covariance.

In the primary analysis, the omnibus ANCOVA indicated a significant overall effect of intervention group on end-of-study HADS total score after adjustment for baseline score, age, sex, and chronic condition (F(2, 688)=25.2; *p* < 0.001; partial ƞ² = 0.068). Pairwise comparisons showed that the eMPower + human support arm significantly improved HADS total score compared with control (mean difference 2.9 points; 95% CI:[2.0, 3.8]; *p* < 0.001), representing a 20% relative improvement (Fig 2, Table 3). Both anxiety and depression subscales significantly improved: anxiety by 1.5 points and depression by 1.4 points (both *p* < 0.001). Control participants showed stable or slightly worsened scores over 12-weeks. Unadjusted between-group comparisons yielded consistent results and are presented in S6 Table.

**Table 3:** Primary and secondary outcomes at 12 weeks: eMPower + human support vs. control (intention-to-treat population).

Outcome	eMPower + human support		Control		Adjusted mean difference (95% CI)	Between-group relative improvement (%)	F(df), p, partial ƞ²
	**Baseline** **mean, (SD)** **min, max** **[valid *n*]**	**12 weeks** **mean, (SD)** **min, max** **[valid *n*]**	**Baseline** **mean, (SD)** **min, max** **[valid *n*]**	**12 weeks** **mean, (SD)** **min, max** **[valid *n*]**			
HADS total	13.5 (6.8); 1, 37; *n* = 276	11.3 (6.2); 0, 39; *n* = 222	13.2 (6.5); 0, 32; *n* = 274	14.2 (7.0); 0, 34; *n* = 259	2.9 (95% CI [2.0, 3.8])	20.4% (95% CI [14.1, 26.8])	F(1,475)=43.7, *p* < 0.001, partial ƞ² = 0.08
HADS-Anxiety	8.0 (4.1); 0, 19; *n* = 276	6.7 (3.6); 0, 21; *n* = 222	7.8 (4.0); 0, 19; *n* = 274	8.2 (4.2); 0, 19; *n* = 259	1.5 (95% CI [1.0, 2.0])	18.3% (95% CI [12.2, 24.4])	F(1,475)=36.9, *p* < 0.001, partial ƞ² = 0.07
HADS-Depression	5.5 (3.6); 0, 18; *n* = 276	4.6 (3.4); 0, 18; *n* = 222	5.4 (3.4); 0, 17; *n* = 274	6.1 (3.7); 0, 19; *n* = 259	1.4 (95% CI [0.9, 1.9])	23.0% (95% CI [14.8, 31.1])	F(1,475)=28.0, *p* < 0.001, partial ƞ² = 0.06
MFIS total	45.8 (17.5); 0, 84; *n* = 276	36.5 (15.8); 0, 82; *n* = 217	44.4 (15.3); 1, 83; *n* = 274	44.7 (16.6); 0, 81; *n* = 258	8.4 (95% CI [6.6, 10.4])	18.8% (95% CI [14.8, 23.3])	F(1,469)=78.9, *p* < 0.001, partial ƞ² = 0.14
MFIS Physical	21.7 (8.2); 0, 36; *n* = 276	17.8 (7.7); 0, 35; *n* = 217	21.5 (7.6); 1, 36; *n* = 274	21.3 (7.9); 0, 36; *n* = 258	3.4 (95% CI [2.5, 4.3])	16.0% (95% CI [11.7, 21.2])	F(1,469)=53.3, *p* < 0.001, partial ƞ² = 0.10
MFIS Cognitive	19.7 (8.9); 0, 40; *n* = 276	15.3 (7.8); 0, 40; *n* = 217	18.7 (7.9); 0, 40; *n* = 274	19.0 (8.6); 0, 40; *n* = 258	4.1 (95% CI [3.1, 5.0])	21.6% (95% CI [16.3, 26.3])	F(1,469)=66.7, *p* < 0.001, partial ƞ² = 0.12
MFIS Psychosocial	4.4 (2.2); 0, 8; *n* = 276	3.4 (2.0); 0, 8; *n* = 217	4.2 (2.0); 0, 8; *n* = 274	4.3 (2.0); 0, 8; *n* = 258	1.0 (95% CI [0.7, 1.2])	23.3% (95% CI [16.3, 27.9])	F(1,469)=43.2, *p* < 0.001, partial ƞ² = 0.08
SF-12 MCS	41.9 (10.0); 19, 63; *n* = 276	50.8 (7.7); 28, 64; *n* = 222	42.7 (10.4); 16, 65; *n* = 274	48.0 (8.3); 27, 67; *n* = 259	3.0 (95% CI [1.8, 4.2])	6.3% (95% CI [3.8, 8.8])	F(1,475)=25.2, *p* < 0.001, partial ƞ² = 0.05
SF-12 PCS	39.1 (10.9); 16, 61; *n* = 276	40.2 (11.9); 6, 61; *n* = 222	39.4 (10.6); 17, 63; *n* = 274	37.6 (12.4); 9, 63; *n* = 259	1.9 (95% CI [0.5, 3.4])	5.1% (95% CI [1.3, 9.0])	F(1,475)=7.27, *p* = 0.009, partial ƞ² = 0.02
EQ-5D-5L VAS	60.4 (19.7); 0, 100; *n* = 276	68.1 (17.7); 15, 99; *n* = 221	63.5 (18.1); 12, 100; *n* = 274	63.7 (18.2); 13, 99; *n* = 259	5.3 (95% CI [2.6, 8.0])	8.3% (95% CI [4.1, 12.6])	F(1,474)=14.6, *p* < 0.001, partial ƞ² = 0.03
EQ-5D-5L Index Score	0.7 (0.2); −0.4, 1; *n* = 276	0.8 (0.2); −0.1, 1; *n* = 221	0.7 (0.2); 0.03, 1; *n* = 274	0.7 (0.2); −0.2, 1; *n* = 259	0.05 (95% CI [0.03, 0.08])	7.1% (95% CI [4.3, 11.4])	F(1,474)=18.7, *p* < 0.001, partial ƞ² = 0.04

Adjusted mean differences and 95% CIs were estimated using ANCOVA adjusting for baseline score, chronic condition type, age, and sex. Valid n reflects participants with available 12-week data. Relative improvement was calculated as the adjusted between-group difference divided by the mean control group score at 12 weeks. Higher HADS and MFIS scores indicate greater symptom burden (positive differences favor the intervention), whereas higher SF-12 and EQ-5D-5L scores indicate better health-related quality of life. EQ-5D-5L index scores range from values <0–1. CI, confidence interval; HADS, Hospital Anxiety and Depression Scale; MFIS, Modified Fatigue Impact Scale; SF-12, Short Form-12; MCS, Mental Component Summary; PCS, Physical Component Summary; VAS, Visual Analog Scale; SD, standard deviation; df, degrees of freedom.

Sensitivity analyses using last observation carried forward (LOCF) for missing 12-week data yielded similar results. The adjusted between-group difference in HADS total score remained significant (2.4; 95% CI [1.7, 3.2]; *p* < 0.001), consistent with the complete-case analysis. Similar findings were observed for HADS anxiety (mean difference 1.3; 95% CI [0.8, 1.7]; *p* < 0.001) and HADS depression (mean difference 1.2; 95% CI [0.7, 1.6]; *p* < 0.001), indicating that results were robust to assumptions regarding missing data (S5 Table).

### Secondary outcomes

**Fig 3:**
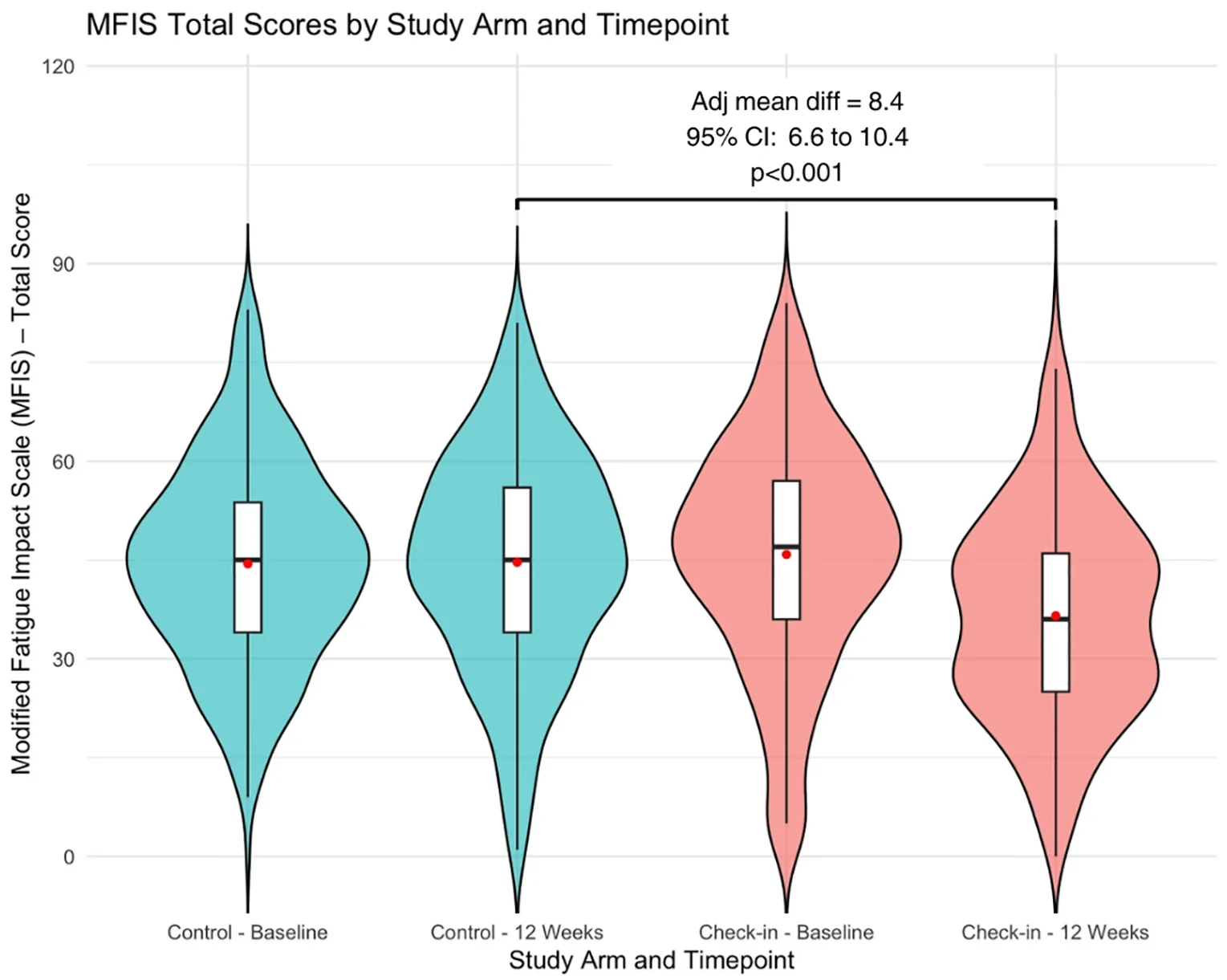
Distribution of fatigue (MFIS total) at baseline and 12 weeks in eMPower + human support vs. waitlist control. Violin plots show the distribution of MFIS total scores at baseline and 12-week follow-up. Blue represents waitlist control and peach represents eMPower + human support. Higher scores indicate greater fatigue severity. Adjusted between-group mean differences with 95% CIs, adjusting for baseline score, chronic condition type, age, and sex, are reported in Table 3. MFIS, Modified Fatigue Impact Scale; ANCOVA, Analysis of covariance.

Participants in the eMPower + human support arm showed improvements in all secondary outcomes compared with control. After Hochberg correction for multiplicity, all six prespecified secondary outcomes remained statistically significant (S7 Table). Under the more conservative Bonferroni correction (α = 0.0083), all apart from the SF-12 PCS score, remained significant. Fatigue showed the largest between-group difference (MFIS total score: 8.4 points; 95% CI [6.6, 10.4]; 19% relative improvement; *p* < 0.001) (Fig 3) with significant between-group differences in physical (16%), cognitive (22%) and psychosocial (23%) domains (all *p* < 0.001).

Mental health-related quality of life (SF-12 MCS) was associated with a 3.0 point between-group difference (95% CI [1.8, 4.2]; *p* < 0.001). Physical health-related quality of life (SF-12 PCS) showed a 1.9-point between-group difference (95% CI [0.5, 3.4]; *p* = 0.009) that remained significant under the Hochberg procedure but narrowly missed the more conservative Bonferroni threshold (α = 0.0083) (Tables 3 and S7). EQ-5D-5L index score was associated with a 0.05 point (*p* < 0.001) between-group difference (Table 3). Unadjusted between-group comparisons yielded consistent results and are presented in S6 Table.

### Prespecified exploratory analyses

#### Combined intervention groups versus control.

In prespecified exploratory analyses, the two intervention arms were combined and compared with control to estimate the overall effect of the eMPower program. Among participants with 12-week data, the combined intervention group (*n* = 436) demonstrated a 2.8-point difference in HADS total score compared with control (*n* = 259) (95% CI [2.0, 3.5]; *p* < 0.001), corresponding to a 20% relative reduction. Significant between-group differences were also observed for HADS anxiety and depression subscales, fatigue (MFIS mean difference 7.8; 95% CI [6.1, 9.5]; *p* < 0.001), and SF-12 MCS score (mean difference 2.9; *p* < 0.001) (S3 Table).

#### Comparison between intervention arms.

In the second prespecified exploratory comparison, no statistically significant differences were observed between intervention arms (*n* = 436 participants with 12-week data; eMPower + human support *n* = 222; self-directed *n* = 214) for any primary or secondary outcomes (all *p* > 0.05; S4 Table).

## Discussion

The eMPower three-arm RCT evaluated a digital, multicomponent intervention for mental health and fatigue in a large, geographically diverse sample of adults with chronic medical conditions. Among 825 participants across 13 countries and more than 20 chronic conditions, both self-directed and human-supported formats produced clinically meaningful improvements in anxiety, depression, fatigue, and quality of life.

Reductions in anxiety and depression were statistically significant (adjusted mean difference 2.9; 95% CI [2.0, 3.8]; *p* < 0.001), with the lower bound of the confidence interval exceeding the established minimum clinically important difference (MCID) of 1.5 to 1.7 points [31], and were comparable to antidepressant trials in chronic disease (standardized mean difference (SMD) ~ 0.3) [32] and structured psychotherapy [33,34]. That a fully digital intervention was associated with differences comparable to both pharmacotherapy and structured psychotherapy highlights its potential clinical utility, particularly given that, unlike pharmacotherapy, this nonpharmacologic intervention avoids medication-related side-effects and does not require intensive clinician involvement. These findings are also consistent with meta-analyses of digital mental health and fatigue interventions showing benefits for depression (SMD –0.33), anxiety (SMD –0.26), and fatigue (SMD –0.74) [8,35].

Direct comparison of self-directed and human-supported formats revealed no significant difference between intervention arms in prespecified exploratory analyses. However, the trial was powered for the primary comparison of eMPower + human support versus control; the absence of a statistically significant difference between arms should therefore not be interpreted as evidence of equivalence. Prior meta-analyses of digital mental health interventions report mixed findings regarding the incremental benefit of human support; although some studies favor supported formats, the overall effect was modest and appears to vary according to factors such as baseline symptom severity, participant demographics, and intervention design [10,36–39]. Several trial-specific factors may help explain the absence of a support effect in this trial. Participants had a range of baseline symptom severity (moderate-severe) and were required to have controlled psychiatric conditions, which likely reduced the proportion of individuals with more severe or treatment-resistant symptoms who may benefit from structured support [10,39]. In addition, support in this trial was brief, nonclinician delivered, and uniformly assigned to a treatment arm rather than targeted to individuals with greater clinical or engagement needs. Evidence from other similar trials suggests that structured and targeted support strategies, particularly those aimed at individuals with low technology proficiency, higher symptom burden, or early nonengagement, may improve adherence and clinical outcomes [37,38]. Taken together, these findings suggest that for anxiety, depression, and fatigue in moderately symptomatic populations, fully digital delivery can achieve clinically meaningful benefits. While these factors were not directly evaluated in the current trial, human support may provide greater value for individuals with higher clinical complexity, lower digital literacy, or in real-world implementation settings where adherence may be lower and stepped-care support can be selectively introduced [40,41].

The integration of psychological and physical techniques in the eMPower cohort likely contributed to between-group differences across both mental and physical outcomes. Fatigue, one of the most debilitating and treatment-resistant symptoms in chronic disease, was associated with a between-group difference of 8.4 (95% CI: 6.6, 10.4) points on the MFIS, surpassing the MCID (4.0 points) [42]. This magnitude of benefit is comparable to conventional nonpharmacological interventions for fatigue, including pulmonary rehabilitation in chronic obstructive pulmonary disease (COPD) [43] and exercise programs in cancer [44], while pharmacological treatments rarely yield sustained improvements in fatigue, even after mental health symptoms improve [45,46].

The eMPower trial findings are consistent with prior meta-analyses of predominantly single-condition studies showing that digital interventions, including self-directed formats, can significantly reduce fatigue [35]. The psychology-based skills likely contributed by fostering psychological flexibility, a relevant technique in chronic illness where uncertainty and symptom persistence are common [47]. Although this trial was not designed to study the isolated effects of individual program components, the results support a synergistic model where psychological and physical approaches work together to address the interrelated physical and mental health burdens of chronic disease [48].

Several considerations strengthen the interpretation of these results. The trial enrolled a large, geographically diverse sample with a broad representation of chronic conditions, including a large proportion of participants classified as frail, individuals often underrepresented in digital health research. By focusing on transferable coping skills rather than symptom suppression alone, the program enhanced the potential for durable benefit across varied conditions and trajectories. Although the trial was not powered for formal condition-specific subgroup analyses, exploratory descriptive patterns across major diagnostic categories appeared directionally consistent with the overall findings [49]. Condition-focused analyses (e.g., digestive disease, liver disease, and chronic kidney disease cohorts) are being reported separately.

Important limitations need to be considered. First, while the chronic disease educational content was tailored by condition, it is likely that further program personalization, and inclusion of other technology features like notifications may further increase the impact. Second, while the 12-week follow-up was sufficient to assess short-term effects, longer-term durability and downstream health outcomes warrant evaluation. Third, human support in this trial was provided by trained, nonclinical personnel, leaving open the question of whether involvement from licensed mental health professionals might yield superior outcomes for those with severe or treatment-resistant symptoms. Fourth, diagnosis verification methods varied across the sample: 39% of participants had diagnoses confirmed through provincial electronic medical records, while the remainder were verified by the study physicians using medication lists and available clinical information. Although randomization would be expected to balance this discrepancy across arms, the absence of standardized diagnostic confirmation for all participants is a limitation. Furthermore, the total number of chronic conditions per participant was not collected, precluding assessment of multimorbidity burden, which may influence treatment response. Fifth, the fully remote design and reliance on self-reported eligibility carry residual risks of undetected duplicate or ineligible participation that cannot be entirely eliminated despite the data integrity procedures employed, including manual review of names and email addresses, orientation attendance tracking, and survey timestamp monitoring. Lastly, the cohort was predominantly female, white, and had a post-secondary education, a pattern consistent with prior digital health studies [20]. The geographic concentration of participants in Canada (81.8%), and Alberta specifically (39%), driven by the study team’s location and patient partner organization networks, may introduce selection bias and limit the generalizability of findings to other regions and healthcare systems.

Taken together, the eMPower clinical trial provides robust evidence that a fully digital, multicomponent intervention reduced anxiety and depression symptoms and was associated with improvements in fatigue and quality of life across a range of chronic disease populations. Rather than developing separate digital programs for each condition, a single cross-condition approach with tailoring for disease-specific education is effective in addressing shared symptoms. Comparable benefits between self-directed and supported formats highlight the feasibility of scalable, low-intensity delivery and reinforces the potential for real-world implementation to meet the needs of a growing population living with chronic conditions.

## Supporting information

S1 Table Behavior change techniques used in the eMPower intervention, mapped to the Behavior Change Technique Taxonomy (v1). (DOCX)

S2 Table Additional baseline demographic, lifestyle, and health status variables by treatment arm. (DOCX)

S3 Table Exploratory analysis comparing combined intervention arms (eMPower + human support and self-directed eMPower) versus control at 12 weeks (intention-to-treat population). (DOCX)

S4 Table Exploratory analysis comparing eMPower + human support versus self-directed eMPower at 12 weeks (intention-to-treat population). (DOCX)

S5 Table Sensitivity analysis using last observation carried forward (LOCF) for missing 12-week outcomes (eMPower + human support versus control). (DOCX)

S6 Table Unadjusted between-group comparisons for primary and secondary outcomes at 12 weeks. (DOCX)

S1 Checklist CONSORT 2025 Checklist. Hopewell S, Chan AW, Collins GS, Hróbjartsson A, Moher D, Schulz KF, and colleagues. CONSORT 2025 Statement: updated guideline for reporting randomised trials. BMJ. 2025;388:e081123. https://dx.doi.org/10.1136/bmj-2024-081123. © 2025 Hopewell and colleagues. This is an Open Access article distributed under the terms of the Creative Commons Attribution License (https://creativecommons.org/licenses/by/4.0/), which permits unrestricted use, distribution, and reproduction in any medium, provided the original work is properly cited.
(DOCX)

S1 File Prespecified statistical analysis plan for the eMPower trial. (DOCX)

S7 Table Multiplicity adjustment for secondary outcomes using Hochberg and Bonferroni corrections: eMPower + human support versus control at 12 weeks (intention-to-treat population). (DOCX)

S2 File Weekly semi-structured check-in script. (DOCX)

S3 File Exemplar weekly intervention newsletter. (PDF)

S4 File eMPower protocol. (DOCX)

S8 Table Disease education video topics. (DOCX)

S2 Checklist CONSERVE-CONSORT checklist for trial modifications. (PDF)

## Abbreviations

ACT: acceptance and commitment therapy
COPD: chronic obstructive pulmonary disease
GDPR: General Data Protection Regulation
HADS: Hospital Anxiety and Depression Scale
LOCF: last observation carried forward
MCID: minimum clinically important difference
MFIS: Modified Fatigue Impact Scale
PBC: Primary Biliary Cholangitis
RCTs: randomized controlled trials
SD: standard deviation
SME: standardized mean difference
VAS: Visual Analog Scale.
