# Supplementary material for: Effect of a digital intervention on mental health symptoms in adults with chronic conditions: A three-arm randomized controlled trial
Source: PLoS Med. 2026 Aug 20;23(8):e1005198. doi: 10.1371/journal.pmed.1005198 (PMC13492819; doi:10.1371/journal.pmed.1005198)
Supplement: Supplementary file 1 [file pmed.1005198.s011.pdf]

Welcome to Week 9!

Updates:

Click here to visit the **EMPOWER** website!

The Benefits of Mindful Movement

We've had a few questions about how the mindful movement program in EMPOWER is different from traditional moderate or high intensity exercise. Without a doubt, both are very beneficial and they can be done alongside each other.

Mindful movement may be new to you and that's ok :), BUT, during your 12-week EMPOWER journey, we would encourage you to fully embrace and explore it. Published studies have identified significant mental health benefits, with reductions in stress, anxiety and depression. Mindful movement also has physical health benefits by increasing flexibility, balance, strength and posture. If you feel that you would benefit from more of a physical challenge during your EMPOWER journey (provided that your healthcare team hasn't told you not to exercise), there are a few options. You can add in some exercise to your day (e.g. a brisk walk) and track it in your tracking log. The tai chi and the yoga can also be made more difficult by carrying out longer or deeper holds, or you can add light weights to the chair program. Please always remember to only do what feels safe and comfortable for you.

Click here to read more on the benefits of mindful movement!

Good Morning...New Meditation!

By mid-week, please keep an eye out for a new Guided Good Morning Meditation! Have a happy week everyone :)

Live Movement Sessions with Patricia

Thank you all so much for your positive feedback of Patricia's live movement sessions and warm-up videos. We are excited to share with you that in December, Patricia will be hosting some live sessions with tai chi-based movements! Please stay tuned for the days and times of these sessions as we finalize them. :)

Quick Links - Table of Contents

We will be adding a new supplemental feature to the Quick Links section! By mid-week, you'll be able to access a table of contents for each program, outlining the life skills content covered in each week. You can use this as a quick reference guide, so you know exactly where to access certain content :)

Group Movement Sessions:

Open to all participants. These online group sessions will be led by our team of movement specialists. Come and move together with other EMPOWERites.

Tuesday, November 29th at 9AM MT

Add event to calendar

Saturday, December 2nd at 10AM MT

Add event to calendar

Click here for Zoom link: Group Movement Sessions

The Importance of Sleep

by Madisyn Taylor

Regular periods of sleep are key to a healthy body and a clear mind, as it is during sleep that your body renews itself.

When life gets busy, sleep is often the first activity that we sacrifice. Considered a luxury by many busy people, sleep is actually as vital to sustaining a balanced life as are breathing, eating, and drinking. Getting sufficient sleep can be a potent energizer, just as not getting enough sleep can leave you feeling drained and sluggish. While eight hours is the average amount of sleep most adults should generally aim for, the right amount of sleep varies for each person. Some people may thrive on just four hours, while others don't feel well rested unless they've slept for ten hours. How much we sleep also varies, depending upon where we are in life. Young people often need more sleep, while older people may need less. The benefits of sleep always stay the same. Regular and consistent periods of wakefulness and sleep are key ingredients to fostering a healthy body and a clear mind. It is during sleep that your body renews itself.

The ability to forgo sleep is considered by some to be an asset. But while it may seem that the nighttime hours can be better used for more productive activities, sleep in itself is extremely productive. During sleep, your body and psyche are both regaining their strength for the coming day. You may even have the unique opportunity to explore the hidden recesses of your personality while you dream. Meanwhile, your long-term memories are reinforced.

Many cultures engage in an afternoon siesta. Taking a nap is refreshing and can increase both productivity and creativity. Author Lewis Carroll is said to have conceived his idea for Alice in Wonderland while dreaming. A good night's sleep also has been known to bring with it the gifts of clarity, wisdom, and a fresh

perspective. Even the ancient Greeks thought of sleep as a gift from the gods. Give yourself the gift of peaceful slumber and you will likely find yourself feeling alert, refreshed, and ready for life's challenges. You may also find yourself feeling more centered, thoughtful, and aware throughout the day so you can live your full potential.

EMPOWER Program

Reprinted from DailyOM – Inspirational thoughts for a happy, healthy and fulfilling day.

If you think you are experiencing any positive or negative side effects after beginning your EMPOWER program, please reach out via email at empower@ualberta.ca.

Copyright © 2023 EMPOWER, All rights reserved.
You are receiving this email as a participant in the EMPOWER study.

Our mailing address is:
EMPOWER
