# Supplementary material for: Effect of a digital intervention on mental health symptoms in adults with chronic conditions: A three-arm randomized controlled trial
Source: PLoS Med. 2026 Aug 20;23(8):e1005198. doi: 10.1371/journal.pmed.1005198 (PMC13492819; doi:10.1371/journal.pmed.1005198)
Supplement: Supplementary file 2 [file pmed.1005198.s014.pdf]

CONSERVE Checklists

Use CONSERVE-CONSORT for completed trial reports and CONSERVE-SPIRIT for trial protocols.

**CONSERVE-CONSORT Extension: 9 June 2026**			
**Item**	**Item Title**	**Description**	**Page No.**
I.	Extenuating Circumstances	No external extenuating circumstances (e.g., pandemic, natural disaster) occurred during the trial. Two investigator-initiated protocol amendments were made to the eligibility criteria to improve inclusivity and equitable access, based on feedback from the trial steering committee, study psychiatrist, and patient partner organizations. This checklist is completed at the editor's request to provide transparency regarding these modifications.	n/a
II.	Important Modifications	a. Two modifications to eligibility criteria were made: (1) The minimum age was lowered from ≥50 to ≥18 years to improve inclusivity and align with the trial's cross-condition design. (2) The HADS-Depression >10 exclusion criterion was removed following consultation with the study psychiatrist and patient partner organizations to improve equitable access, while retaining exclusion for near-daily suicidality and uncontrolled psychiatric illness. These are important modifications because they changed the eligible study population.	Methods, “Study design and participant,” paragraph 3
		b. See CONSORT items below	(see below)
		c. Amendment 1: Approved and implemented May 19, 2023 (180 of 825 participants enrolled, 21.8%)<br>Amendment 2: Approved and implemented October 21, 2023 (321 of 825 participants enrolled, 38.9%)	Methods, “Study design and participant”, paragraph 3
III.	Responsible Parties	Modifications were proposed by the trial steering committee (study statistician, principal investigator, and three clinical team members), reviewed in consultation with the study psychiatrist and patient partner organizations, and approved by the University of Alberta Health Research Ethics Board prior to implementation.	Methods, “Study design and participant,’ paragraph 3

IV.	Interim data	If modifications were informed by trial data, describe how the interim data were used, including whether they were examined by study group, and whether the individuals reviewing the data were blinded to the treatment allocation.			n/a
**CONSORT Number and Item**		No interim efficacy analyses were conducted. Modifications were based on inclusivity, feasibility, and clinical safety considerations, not on accumulating outcome data. No trial data were examined by study group, and no unblinding occurred.			**Page No.**
		**No Change**	**Impact***	**Mitigating Strategy****	
1	Title and abstract			Abstract describes the final eligibility criteria as implemented.	Abstract
2	Introduction				Introduction
3	Methods: Trial Design				Methods, "Study design and participants," paragraph 2 and 3

4	Methods: Participants			Impact: Eligibility criteria were modified (age lowered from ≥50 to ≥18; HADS-D >10 exclusion removed), expanding the eligible population. Mitigating strategy: Stratified block randomization by chronic condition maintained balance across arms. Safety maintained through retained exclusions for uncontrolled psychiatric illness and near-daily suicidality. Both amendments described with dates, enrollment counts, rationale, and ethics approval.	Methods, "Study design and participants," paragraph 2-4
5	Methods: Interventions				Methods, "Interventions"
6	Methods: Outcomes				Methods, "Outcomes"
7	Methods: Sample Size				Methods, "Sample size and statistical analysis"
8-10	Methods: Randomisation				Methods, "Randomization and masking"
11	Methods: Blinding				Methods, "Randomization and masking"

12	Methods: Statistical methods				Methods, "Sample size and statistical analysis"
13	Results: Participant flow			CONSORT flow diagram (Fig 1) reflects final eligibility criteria; amendment details provided in Methods.	Results, "Participant flow"; Fig 1
14	Results: Recruitment				Results, "Participant flow and baseline characteristics," paragraph 1
15	Results: Baseline data				Results, "Participant flow and baseline characteristics"; paragraph 2, table 1
16	Results: Numbers analysed				Results; Table 3
	Results: Outcomes and estimation				Results, "Primary outcome" and "Secondary outcomes"
18	Results: Ancillary analyses				Results, "Pre-specified exploratory analyses"
19	Results: Harms				Results, "Participant flow and baseline characteristics," paragraph 1

20	Discussion: Limitations			Limitations section acknowledges sample characteristics, generalizability, and diagnosis verification.	Discussion, paragraph 5
21	Discussion: Generalisability			Generalizability discussed within limitations, including geographic concentration and demographic composition.	Discussion, paragraph 7
	Other information: Registration			ClinicalTrials.gov record updated to reflect both amendments.	Methods, "Study design and participants," paragraph 1
24	Other information: Protocol			Published protocol (Johnson et al., 2024) details both amendments.	Methods, "Study design and participants," paragraph 4; Reference 12
25	Other information: Funding				Title page

*Aspects of the trial that are directly affected or changed by the extenuating circumstance and are not under the control of investigators, sponsor or funder.
**Aspects of the trial that are modified by the study investigators, sponsor or funder to respond to the extenuating circumstance or manage the direct impacts on the trial.
