# Supplementary material for: Effect of a digital intervention on mental health symptoms in adults with chronic conditions: A three-arm randomized controlled trial
Source: PLoS Med. 2026 Aug 20;23(8):e1005198. doi: 10.1371/journal.pmed.1005198 (PMC13492819; doi:10.1371/journal.pmed.1005198)
Supplement: Supplementary file 3 [file pmed.1005198.s001.docx]

**S1 Table. Behaviour change techniques used in the eMPower intervention mapped to the BCCTv1**

| **BCT group** | **BCT label** | **Application in eMPower** |
| --- | --- | --- |
| **1. Goals and planning** | 1.1 Goal setting (behaviour) | - Participants encouraged to set weekly self-care goals through reflective exercises and check-ins (for those in empower + human support arm) - Participants were encouraged to engage in approximately 90 minutes of content per week - In week 3, participants were advised to set a specific, measurable, achievable, relevant and time-bound (SMART) goal for a health behaviour in their life |
|  | 1.4 Action planning | - All participants received an orientation to the online platform via Zoom, where the research team provided information and a demonstration about using the program. This was followed by written content and a webpage with the pre-recorded version of this demonstration - Participants watched an interactive video focused on goal setting related to the program, including frequency of program engagement, days of the week, and times - The platform contained an optional weekly calendar to select days/times of the week participants intended to engage in the program |
|  | 1.5 Review behaviour goal(s) | - Participants’ weekly adherence vs target adherence goal was discussed during check in (for those in empower + human support arm) |
|  | 1.6 Discrepancy between current behaviour and goal | - During week 12, participants were invited to revisit their SMART goal and adjust as needed |
| **2. Feedback and monitoring** | 2.1 Monitoring of behaviour by others without feedback | - The online platform recorded days participants were active in the program, revealing a ‘streak’ for the number of days in a row |
|  | 2.2 Feedback on behaviour | - Participants received points for the number of minutes they engaged with the program practices (e.g., 10-minute ACT-informed curriculum video = 10 points) |
|  | 2.3 Self-monitoring of behaviour | - An optional weekly journal allowed participants to track and receive points for movement they engaged in outside of the program (group sessions and personal movement) |
| **3. Social support** | 3.1 Social support (unspecified)  3.3 Social support (emotional) | - The weekly check-in (for those in empower + human support arm) offered non-clinician support to sustain engagement - Check-in staff were trained in motivational interviewing and supervised weekly by a clinical psychologist - Participants were invited to join two optional group sessions that included program practices facilitated by a team member and shared with peers - The weekly check-ins fostered connection, encouragement, and nonjudgmental support (for those in empower + human support arm) |
| **4. Shaping knowledge** | 4.1 Instruction on how to perform the behaviour | - Educational guided videos and optional group sessions taught techniques like breathwork, meditation, and pacing - Educational videos by a clinical psychologist (DLB) provided information and practical tips for implementing ACT techniques into daily life - Modifications were provided for mindful movement (three types of movement, different lengths) and breathwork activities. - The optional weekly group sessions offered participants an opportunity to seek clarity on specific movement, breathwork, or meditation techniques |
| **5. Natural consequences** | 5.1 Information about health consequences | - Content explained how stress, fatigue, and inactivity relate to chronic condition burden - Weekly condition-specific videos discussed topics like diet, protein intake, disease complications, etc. |
|  | 5.2 Information about emotional consequences | - Weekly chronic disease coping skills videos from the ACT-informed curriculum provided information about emotional distress, causes, rationale, and techniques to cope |
| **6. Comparison of behaviour** | 6.1 Demonstration of behaviour | - All participants received an orientation to the online platform via Zoom, where the research team provided information and demonstrated how to use the program. This was supplemented by written instruction and a webpage with the pre-recorded version of this demonstration - Guided videos were provided demonstrating how to perform the mindful movement, breathwork, and select meditation practices. Accommodations were also provided - Testimonials, quotes, and examples showed how others used the program to improve wellbeing (shared via weekly newsletter) |
|  | 6.2 Social comparison | - All participants were invited to attend weekly live group movement sessions via Zoom |
| **7. Associations** | 7.1 Prompts/cues | - Weekly emails (at the outset of the new week) reminded participants to engage with new content and practices |
| **8. Repetition and substitution** | 8.1 Behavioural practice/rehearsal  8.3 Habit formation | - Encouraged repeated practice of mindful movement, meditation, and breathing exercises - Each week of the intervention followed a consistent structure, featuring the same core content types and approximate duration. Participants received a ~10-minute weekly module comprising ACT-informed curriculum, guided somatic practices (e.g., meditation, breathwork, mindful movement), and tailored condition-specific education |
|  | 8.7 Graded tasks | - Weekly mind -body practices progressed week by week |
| **9. Comparison of outcomes** | 9.1 Credible source | - Weekly condition-specific videos were provided by specialists - The ACT-informed curriculum was developed and delivered by a clinical psychologist (DLB) - The mind-body content was developed and delivered by expert teams |
| **11. Regulation** | 11.2 Reduce negative emotions | - ACT-informed curriculum and somatic practices supported emotional regulation and distress tolerance |
| **13. Identity** | 13.1 Identification of self as role model | - Encouraged empowerment and framing of self-care as identity-consistent behaviour |
| **15. Self-belief** | 15.1 Verbal persuasion about capability | - The program content, weekly check-ins, and weekly emails emphasized that small consistent actions could lead to meaningful change |

Behaviour change techniques (BCTs) are categorized according to the Behaviour Change Technique Taxonomy v1 (BCCTv1); Each technique is described with its specific application within the eMPower program. BCT = behaviour change technique; ACT = Acceptance and Commitment Therapy; SMART = specific, measurable, achievable, relevant, and time-bound
