# Supplementary material for: Effect of a digital intervention on mental health symptoms in adults with chronic conditions: A three-arm randomized controlled trial
Source: PLoS Med. 2026 Aug 20;23(8):e1005198. doi: 10.1371/journal.pmed.1005198 (PMC13492819; doi:10.1371/journal.pmed.1005198)
Supplement: Supplementary file 4 [file pmed.1005198.s002.docx]

**S2 Table. Additional baseline demographic, lifestyle, and health status variables by treatment arm**

|  | **Total (n=825)** | **eMPower + human support**  **(n=276)** | **eMPower (n=275)** | **Control (n=274)** |
| --- | --- | --- | --- | --- |
| **Continent,** n (%) | | | | |
| North America | 789 (95.6%) | 267 (96.7%) | 258 (93.8%) | 264 (96.4%) |
| South America | 1 (0.1%) | — | — | 1 (0.4%) |
| Europe | 18 (2.2%) | 6 (2.2%) | 6 (2.2%) | 6 (2.2%) |
| Australia | 5 (0.6%) | — | 3 (1.1%) | 2 (0.7%) |
| Asia | 3 (0.4%) | 1 (0.4%) | 1 (0.4%) | 1 (0.4%) |
| Prefer not to answer or other | 9 (1.1%) | 2 (0.7%) | 7 (2.5%) | — |
| **Baseline technology proficiency,** n (%) | | | | |
| Not proficient/confident | 5 (0.6%) | 3 (1.1%) | 1 (0.4%) | 1 (0.4%) |
| A little proficient/confident | 18 (2.2%) | 5 (1.8%) | 5 (1.8%) | 8 (2.9%) |
| Somewhat proficient/confident | 41 (5.0%) | 14 (5.1%) | 18 (6.5%) | 9 (3.3%) |
| Moderately proficient/confident | 278 (33.7%) | 92 (33.3%) | 81 (29.5%) | 105 (38.3%) |
| Highly proficient/confident | 329 (39.9%) | 110 (39.9%) | 114 (41.5%) | 105 (38.3%) |
| Technology expert | 154 (18.7%) | 52 (18.8%) | 56 (20.4%) | 46 (16.8%) |
| **Meditation experience in the last 6 months** | | | | |
| Yes, n (%) | 331 (40.1%) | 106 (38.4%) | 112 (40.7%) | 113 (41.2%) |
| **Yoga experience in the last 6 months** | | | | |
| Yes, n (%) | 224 (27.2%) | 73 (26.4%) | 77 (28.0%) | 74 (27.0%) |
| **Stress level at home (0=no stress, 10=most stressed),** mean (SD) | 4.9 (2.3) | 5.0 (2.3) | 4.9 (2.3) | 4.8 (2.3) |
| **Primary earner,** n (%) | | | | |
| Yes | 407 (49.3%) | 136 (49.3%) | 135 (49.1%) | 136 (49.6%) |
| **Primary person responsible for housework,** n (%) | | | | |
| Yes | 599 (72.6%) | 202 (73.2%) | 204 (74.2%) | 193 (70.4%) |
| **Number of people living in the home** (mean, SD) | 1.78 (1.31) | 1.77 (1.32) | 1.74 (1.33) | 1.84 (1.30) |
| **EQ-5D-5L**  *Mobility* | | | | |
| No problems, n (%) | 421 (51.0%) | 140 (51.1%) | 143 (52.0%) | 138 (50.0%) |
| Slight problems, n (%) | 207 (25.1%) | 74 (27.0%) | 67 (24.4%) | 66 (23.9%) |
| Moderate problems, n (%) | 146 (17.7%) | 43 (15.7%) | 54 (19.6%) | 49 (17.8%) |
| Severe problems, n (%) | 48 (5.8%) | 16 (5.8%) | 11 (4.0%) | 21 (7.6%) |
| Unable/Extreme problems, n (%) | 3 (0.4%) | 1 (0.4%) | — | 2 (0.7%) |
| *Self care* | | | | |
| No problems, n (%) | 664 (80.5%) | 218 (79.6%) | 221 (80.4%) | 225 (81.5%) |
| Slight problems, n (%) | 99 (12.0%) | 38 (13.9%) | 32 (11.6%) | 29 (10.5%) |
| Moderate problems, n (%) | 53 (6.4%) | 16 (5.8%) | 17 (6.2%) | 20 (7.2%) |
| Severe problems, n (%) | 7 (0.8%) | 2 (0.7%) | 4 (1.5%) | 1 (0.4%) |
| Unable/Extreme problems, n (%) | 2 (0.2%) | — | 1 (0.4%) | 1 (0.4%) |
| *Usual activities* | | | | |
| No problems, n (%) | 249 (30.2%) | 87 (31.8%) | 76 (27.6%) | 86 (31.2%) |
| Slight problems, n (%) | 298 (36.1%) | 99 (36.1%) | 108 (39.3%) | 91 (33.0%) |
| Moderate problems, n (%) | 195 (23.6%) | 63 (23.0%) | 64 (23.3%) | 68 (24.6%) |
| Severe problems, n (%) | 67 (8.1%) | 19 (6.9%) | 24 (8.7%) | 24 (8.7%) |
| Unable/Extreme problems, n (%) | 16 (1.9%) | 6 (2.2%) | 3 (1.1%) | 7 (2.5%) |
| *Pain* | | | | |
| No problems, n (%) | 109 (13.2%) | 38 (13.9%) | 32 (11.6%) | 39 (14.1%) |
| Slight problems, n (%) | 331 (40.1%) | 114 (41.6%) | 117 (42.5%) | 100 (36.2%) |
| Moderate problems, n (%) | 280 (33.9%) | 96 (35.0%) | 85 (30.9%) | 99 (35.9%) |
| Severe problems, n (%) | 87 (10.5%) | 20 (7.3%) | 37 (13.5%) | 30 (10.9%) |
| Unable/Extreme problems, n (%) | 18 (2.2%) | 6 (2.2%) | 3 (1.5%) | 8 (2.9%) |
| *Anxiety/depression* | | | | |
| No problems, n (%) | 185 (22.4%) | 58 (21.0%) | 62 (22.5%) | 65 (23.7%) |
| Slight problems, n (%) | 338 (41.0%) | 114 (41.6%) | 114 (41.5%) | 110 (39.9%) |
| Moderate problems, n (%) | 259 (31.4%) | 83 (30.3%) | 80 (29.1%) | 96 (34.8%) |
| Severe problems, n (%) | 39 (4.7%) | 10 (3.6%) | 18 (6.5%) | 11 (4.0%) |
| Unable/Extreme problems, n (%) | 4 (0.5%) | 2 (0.7%) | 1 (0.4%) | 1 (0.4%) |

Data are presented as mean (SD) for continuous variables and n (%) for categorical variables; EQ-5D-5L responses reflect baseline self-reported health status across five domains; SD = standard deviation; EQ-5D-5L = EuroQol 5-Dimension 5-Level; — indicates zero participants
