# Supplementary material for: Effect of a digital intervention on mental health symptoms in adults with chronic conditions: A three-arm randomized controlled trial
Source: PLoS Med. 2026 Aug 20;23(8):e1005198. doi: 10.1371/journal.pmed.1005198 (PMC13492819; doi:10.1371/journal.pmed.1005198)
Supplement: Supplementary file 5 [file pmed.1005198.s003.docx]

**S3 Table. Exploratory analysis comparing combined intervention arms versus control at 12 weeks (intention-to-treat population)**

|  | **Combined eMPower arms** | | **Control** | | **Adjusted mean difference (95% CI)** | **Between-group relative improvement (%) (95% CI)** | **F(df), p, partial ƞ²** |
| --- | --- | --- | --- | --- | --- | --- | --- |
|  | **Baseline**  **mean, (SD),** **min, max**  **[valid n]** | **12 weeks**  **mean, (SD)**, **min, max**  **[valid n]** | **Baseline**  **mean, (SD)**, **min, max**  **[valid n]** | **12 weeks**  **mean, (SD)**, **min, max**  **[valid n]** |  |  |  |
| **Primary outcome:** | | | | | | | |
| HADS total score | 13.6 (6.5); 0, 37; n=551 | 11.6 (6.5); 0, 39; n=436 | 13.2 (6.5); 0, 32; n=274 | 14.2 (7.0); 0, 34; n=259 | 2.8 (95% CI [2.0, 3.5]) | 19.7% (95% CI [14.1, 24.6]) | F(1,689)=50.0, p<0.001, partial ƞ²=0.07 |
| **Secondary outcomes:** | | | | | | | |
| HADS-Anxiety | 8.1 (4.0); 0, 19; n=551 | 6.9 (3.7); 0, 21; n=436 | 7.8 (4.0); 0, 19; n=274 | 8.2 (4.2); 0, 19; n=259 | 1.4 (95% CI [0.9, 1.8]) | 17.1% (95% CI [11.0, 22.0]) | F(1,698)=39.1, p<0.001, partial ƞ²=0.05 |
| HADS-Depression | 5.5 (3.6); 0, 18; n=551 | 4.7 (3.6); 0, 18; n=436 | 5.4 (3.4); 0, 17; n=274 | 6.1 (3.7); 0, 19; n=259 | 1.4 (95% CI [0.9, 1.8]) | 23.0% (95% CI [14.8, 29.5]) | F(1,689)=36.1, p<0.001, partial ƞ²=0.05 |
| MFIS Total | 45.9 (16.3); 0, 84; n=551 | 37.4 (16.2); 0, 82; n=426 | 44.4 (15.3); 1, 83; n=274 | 44.7 (16.6); 0, 81; n=258 | 7.8 (95% CI [6.1, 9.5]) | 17.4% (95% CI [13.6, 21.3]) | F(1,678)=82.1, p<0.001, partial ƞ²=0.10 |
| MFIS Physical | 21.9 (7.9); 0, 36; n=551 | 18.2 (8.0); 0, 36; n=426 | 21.5 (7.6); 1, 36; n=274 | 21.3 (7.9); 0, 36; n=258 | 3.2 (95% CI [2.4, 4.0]) | 15.0% (95% CI [11.3, 18.8]) | F(1,678)=58.6, p<0.001, partial ƞ²=0.08 |
| MFIS Cognitive | 19.6 (8.2); 0, 40; n=551 | 15.7 (7.9); 0, 40; n=426 | 18.7 (7.9); 0, 40; n=274 | 19.0 (8.6); 0, 40; n=258 | 3.7 (95% CI [2.9, 4.6]) | 19.5% (95% CI [15.3, 24.2]) | F(1,678)=72.2, p<0.001, partial ƞ²=0.10 |
| MFIS Psychosocial | 4.4 (2.1); 0, 8; n=551 | 3.5 (2.0); 0, 8; n=426 | 4.2 (2.0); 0, 8; n=274 | 4.3 (2.0); 0, 8; n=258 | 0.9 (95% CI [0.6, 1.1]) | 20.9% (95% CI [14.0, 25.6]) | F(1,678)=49.7, p<0.001, partial ƞ²=0.07 |
| SF-12 MCS | 41.9 (10.3); 15, 65; n=551 | 50.8 (8.0); 25, 70; n=435 | 42.7 (10.4); 16, 65; n=274 | 48.0 (8.3); 27, 67; n=259 | 2.9 (95% CI [1.8, 4.0]) | 6.0% (95% CI [3.8, 8.3]) | F(1,688)=28.9, p<0.001, partial ƞ²=0.04 |
| SF-12 PCS | 39.1 (10.7); 11, 61; n=551 | 39.9 (12.1); 6, 62; n=435 | 39.4 (10.6); 17, 63; n=274 | 37.6 (12.4); 9, 63; n=259 | 2.0 (95% CI [0.8, 3.3]) | 5.3% (95% CI [2.1, 8.8)] | F(1,688)=10.6, p=0.001; partial ƞ²=0.02 |
| EQ-5D-5L VAS | 60.6 (19.1); 0, 100; n=551 | 67.9 (17.6); 15, 99; n=434 | 63.5 (18.1); 12, 100; n=274 | 63.7 (18.2); 13, 99; n=259 | 5.0 (95% CI [2.8, 7.3]) | 7.8% (95% CI [4.4, 11.5]) | F(1,687)=19.1, p<0.001, partial ƞ²=0.03 |
| EQ-5D-5L Index Score | 0.7 (0.2); -0.4, 1; n=551 | 0.8 (0.2); -0.2, 1; n=434 | 0.7 (0.2); -0.03, 1; n=274 | 0.7 (0.2); -0.2, 1; n=259 | 0.05 (95% CI [0.03, 0.07]) | 7.1% (95% CI [4.3, 10.0]) | F(1,687)=27.3, p<0.001, partial ƞ²=0.04 |

Adjusted mean differences and 95% CIs were estimated using ANCOVA adjusting for baseline score, chronic condition type, age, and sex. Relative improvement was calculated as the adjusted between-group difference divided by the mean control group score at 12 weeks. Positive adjusted differences favor the intervention for all outcomes. EQ-5D-5L index scores range from values <0 to 1. ANCOVA = analysis of covariance; CI = confidence interval; HADS = Hospital Anxiety and Depression Scale; MFIS = Modified Fatigue Impact Scale; SF-12 = Short Form-12; MCS = Mental Component Summary; PCS = Physical Component Summary; VAS = Visual Analog Scale; SD = standard deviation; df = degrees of freedom.
