# Supplementary material for: Effect of a digital intervention on mental health symptoms in adults with chronic conditions: A three-arm randomized controlled trial
Source: PLoS Med. 2026 Aug 20;23(8):e1005198. doi: 10.1371/journal.pmed.1005198 (PMC13492819; doi:10.1371/journal.pmed.1005198)
Supplement: Supplementary file 6 [file pmed.1005198.s004.docx]

**S4 Table. Exploratory analysis comparing eMPower + human support versus self-directed eMPower at 12 weeks (intention-to-treat population)**

|  | **eMPower + human support** | | **eMPower** | | **Adjusted mean difference (95% CI)** | **Between-roup relative improvement (%) (95% CI)** | **F(df), p, partial ƞ²** |
| --- | --- | --- | --- | --- | --- | --- | --- |
|  | **Baseline**  **mean (SD)**, **min, max**  **[valid n]** | **12 weeks**  **mean(SD, min, max**  **[valid n]** | **Baseline**  **mean, (SD)**, **min, max**  **[valid n]** | **12 weeks**  **mean, (SD)**, **min, max**  **[valid n]** |  |  |  |
| **Primary outcome:** | | | | | | | |
| HADS total score | 13.5 (6.8); 1, 37; n=276 | 11.3 (6.2); 0, 39; n=222 | 13.7 (6.2); 0, 34; n=275 | 11.9 (6.7); 0, 36; n=214 | 0.3 (95% CI [-0.6, 1.3]) | 2.5% (95% CI [-5.0, 10.9]) | F(1,430)=0.5, p=0.409, partial ƞ²=0.001 |
| **Secondary outcomes:** | | | | | | | |
| HADS-Anxiety | 8.0 (4.1); 0, 19; n=276 | 6.7 (3.6); 0, 21; n=222 | 8.1 (3.8); 0, 19; n=275 | 7.2 (3.8); 0, 19; n=214 | 0.4 (95% CI [-0.2, 0.9]) | 5.6% (95% CI [-2.8, 12.5]) | F(1,430)=1.7, p=0.192, partial ƞ²=0.004 |
| HADS-Depression | 5.5 (3.6); 0, 18; n=276 | 4.6 (3.4); 0, 18; n=222 | 5.5 (3.6); 0, 18; n=275 | 4.7 (3.7); 0, 18; n=214 | 0.03 (95% CI [-0.5, 0.6]) | 0.6% (95% CI [-10.6, 12.8]) | F(1,430)=0.01, p=0.916, partial ƞ²<0.001 |
| MFIS Total | 45.8 (17.5); 0, 84; n=276 | 36.5 (15.8); 0, 82; n=217 | 46.0 (14.9); 9, 79; n=275 | 38.3 (16.6); 3, 82; n=209 | 1.3 (95% CI [-0.9, 3.4]) | 3.4% (95% CI [-2.3, 8.9]) | F(1,420)=1.3, p=0.249, partial ƞ²=0.003 |
| MFIS Physical | 21.7 (8.2); 0, 36; n=276 | 17.8 (7.7); 0, 35; n=217 | 22.1 (7.7); 1, 36; n=275 | 18.7 (8.3); 0, 36; n=209 | 0.5 (95% CI [-0.5, 1.5]) | 2.7% (95% CI [-2.7, 8.0]) | F(1,420)=0.8, p=0.364, partial ƞ²=0.002 |
| MFIS Cognitive | 19.7 (8.9); 0, 40; n=276 | 15.3 (7.8); 0, 40; n=217 | 19.5 (7.4); 2, 40; n=275 | 16.0 (8.0); 0, 40; n=209 | 0.6 (95% CI [-0.4, 1.7]) | 3.8% (95% CI [-2.5, 10.6]) | F(1,420)=1.3, p=0.252, partial ƞ²=0.003 |
| MFIS Psychosocial | 4.4 (2.2); 0, 8; n=276 | 3.4 (2.0); 0, 8; n=217 | 4.4 (2.0); 0, 8; n=275 | 3.6 (2.0); 0, 8; n=209 | 0.2 (95% CI [-0.2, 0.5]) | 5.6% (95% CI [-5.6, 13.9]) | F(1,420)=1.0, p=0.327, partial ƞ²=0.002 |
| SF-12 MCS | 41.9 (10.0); 19, 63; n=276 | 50.8 (7.7); 28, 64; n=222 | 42.0 (10.5); 15, 65; n=275 | 50.8 (8.3); 25, 70; n=213 | 0.1 (95% CI [-1.5, 1.2]) | 0.2% (95% CI [-3.0, 2.4]) | F(1,429)=0.04, p=0.848, partial ƞ²<0.001 |
| SF-12 PCS | 39.1 (10.9); 16, 61; n=276 | 40.2 (11.9); 6, 61; n=222 | 39.0 (10.5); 11, 59; n=275 | 39.5 (12.4); 6, 62; n=213 | 0.2 (95% CI [-1.4, 1.7]) | 0.5% (95% CI [-3.5, 4.3]) | F(1,429)=0.04, p=0.841, partial ƞ²<0.001 |
| EQ-5D-5L VAS | 60.4 (19.7); 0, 100; n=276 | 68.1 (17.7); 15, 99; n=221 | 60.8 (18.6); 18, 99; n=275 | 67.7 (17.5); 20, 97; n=213 | -0.6 (95% CI [-3.4, 2.2]) | -0.9% (95% CI [-5.0, 3.2]) | F(1,428)=0.2, p=0.694, partial ƞ²<0.001 |
| EQ-5D-5L Index Score | 0.7 (0.2); -0.4, 1; n=276 | 0.8 (0.2); -0.1, 1; n=221 | 0.7 (0.2); -0.4, 1; n=275 | 0.8 (0.2); -0.2, 1; n=213 | 0.001 (95% CI [-0.02, 0.02]) | 0.1% (95% CI [-2.5, 2.5]) | F(1,428)=0.004, p=0.949, partial ƞ²<0.001 |

Adjusted mean differences and 95% CIs were estimated using ANCOVA adjusting for baseline score, chronic condition type, age, and sex. Relative improvement was calculated as the adjusted between-group difference divided by the mean self-directed eMPower group score at 12 weeks. Positive adjusted differences favor the eMPower + human support group. EQ-5D-5L index scores range from values <0 to 1. ANCOVA = analysis of covariance; CI = confidence interval; HADS = Hospital Anxiety and Depression Scale; MFIS = Modified Fatigue Impact Scale; SF-12 = Short Form-12; MCS = Mental Component Summary; PCS = Physical Component Summary; VAS = Visual Analog Scale; SD = standard deviation; df = degrees of freedom.
