# Supplementary material for: Effect of a digital intervention on mental health symptoms in adults with chronic conditions: A three-arm randomized controlled trial
Source: PLoS Med. 2026 Aug 20;23(8):e1005198. doi: 10.1371/journal.pmed.1005198 (PMC13492819; doi:10.1371/journal.pmed.1005198)
Supplement: Supplementary file 7 [file pmed.1005198.s005.docx]

**S5 Table. Sensitivity analysis using last observation carried forward (LOCF) for missing 12-week outcomes (eMPower + human support versus control)**

| **Outcome** | **eMPower + human support (12 weeks), mean (SD) [n]** | **Control, mean (SD) [n]** | **Adjusted mean difference (95% CI)** | **F(df), p-value, partial ƞ²** |
| --- | --- | --- | --- | --- |
| HADS-Total | 12.1 (6.7) [n=276] | 14.3 (7.1) [n=274] | 2.4 (95% CI [1.7, 3.2]) | F(1,544)=38.4, p<0.001, partial ƞ²=0.07 |
| HADS-Anxiety | 7.1 (3.9) [n=276] | 8.2 (4.2) [n=274] | 1.3 (95% CI [0.8, 1.7]) | F(1,544)=30.8, p<0.001, partial ƞ²=0.05 |
| HADS-Depression | 5.0 (3.7) [n=276] | 6.1 (3.7) [n=274] | 1.2 (95% CI [0.7, 1.6]) | F(1,544)=25.4, p<0.001, partial ƞ²=0.05 |

Baseline values were carried forward for participants with missing 12-week data, yielding the full randomized sample (n=276 intervention, n=274 control). Adjusted mean differences and 95% CIs were estimated using ANCOVA adjusting for baseline score, chronic condition type, age, and sex. Higher HADS scores indicate greater symptom burden (positive adjusted differences favor the intervention). HADS = Hospital Anxiety and Depression Scale; SD = standard deviation; ANCOVA = analysis of covariance; CI = confidence interval; df = degrees of freedom
