# Supplementary material for: Effect of a digital intervention on mental health symptoms in adults with chronic conditions: A three-arm randomized controlled trial
Source: PLoS Med. 2026 Aug 20;23(8):e1005198. doi: 10.1371/journal.pmed.1005198 (PMC13492819; doi:10.1371/journal.pmed.1005198)
Supplement: Supplementary file 8 [file pmed.1005198.s006.docx]

**S6 Table. Unadjusted between-group comparisons for primary and secondary outcomes at 12 weeks**

| Comparison | Outcome | Intervention (12weeks) mean (SD) [n] | Comparator (12 weeks) mean (SD) [n] | Unadjusted mean difference (95% CI) | p-value |
| --- | --- | --- | --- | --- | --- |
| eMPower + human support versus control | | | | | |
| HADS Total | | 11.3 (6.2) [n=222] | 14.2 (7.0) [n=259] | 2.9 (95% CI [1.8, 4.1]) | p<0.001 |
| HADS-Anxiety | | 6.7 (3.6) [n=222] | 8.2 (4.2) [n=259] | 1.5 (95% CI [0.8, 2.2]) | p<0.001 |
| HADS-Depression | | 4.6 (3.4) [n=222] | 6.1 (3.7) [n=259] | 1.4 (95% CI [0.8, 2.1]) | p<0.001 |
| MFIS Total | | 36.5 (15.8) [n=217] | 44.7 (16.6) [n=258] | 8.1 (95% CI [5.2, 11.1]) | p<0.001 |
| MFIS Physical | | 17.8 (7.7) [n=217] | 21.3 (7.9) [n=258] | 3.5 (95% CI [2.1, 5.0]) | p<0.001 |
| MFIS Cognitive | | 15.3 (7.8) [n=217] | 19.0 (8.6) [n=258] | 3.7 (95% CI [2.2, 5.2]) | p<0.001 |
| MFIS Psychosocial | | 3.4 (2.0) [n=217] | 4.3 (2.0) [n=258] | 0.9 (95% CI [0.5, 1.2]) | p<0.001 |
| SF-12 MCS | | 50.8 (7.7) [n=222] | 48.0 (8.3) [n=259] | 2.8 (95% CI [1.4, 4.2]) | p<0.001 |
| SF-12 PCS | | 40.2 (11.9) [n=222] | 37.6 (12.4) [n=259] | 2.6 (95% CI [0.4, 4.8]) | p=0.020 |
| EQ-5D-5L VAS | | 68.1 (17.7) [n=221] | 63.7 (18.2) [n=259] | 4.5 (95% CI [1.2, 7.7]) | p=0.007 |
| EQ-5D-5L Index | | 0.78 (0.17) [n=221] | 0.72 (0.21) [n=259] | 0.05 (95% CI [0.02, 0.09]) | p=0.002 |
| Combined interventions versus control | | | | | |
| HADS Total | | 11.6 (6.5) [n=436] | 14.2 (7.0) [n=259] | 2.7 (95% CI [1.6, 3.7]) | p<0.001 |
| HADS-Anxiety | | 6.9 (3.7) [n=436] | 8.2 (4.2) [n=259] | 1.3 (95% CI [0.7, 1.9]) | p<0.001 |
| HADS-Depression | | 4.7 (3.6) [n=436] | 6.1 (3.7) [n=259] | 1.4 (95% CI [0.8, 1.9]) | p<0.001 |
| MFIS Total | | 37.4 (16.2) [n=426] | 44.7 (16.6) [n=258] | 7.3 (95% CI [4.7, 9.8]) | p<0.001 |
| MFIS Physical | | 18.2 (8.0) [n=426] | 21.3 (7.9) [n=258] | 3.1 (95% CI [1.9, 4.3]) | p<0.001 |
| MFIS Cognitive | | 15.7 (7.9) [n=426] | 19.0 (8.6) [n=258] | 3.4 (95% CI [2.1, 4.6]) | p<0.001 |
| MFIS Psychosocial | | 3.5 (2.0) [n=426] | 4.3 (2.0) [n=258] | 0.8 (95% CI [0.5, 1.1]) | p<0.001 |
| SF-12 MCS | | 50.8 (8.0) [n=435] | 48.0 (8.3) [n=259] | 2.8 (95% CI [1.6, 4.1]) | p<0.001 |
| SF-12 PCS | | 39.9 (12.1) [n=435] | 37.6 (12.4) [n=259] | 2.2 (95% CI [0.4, 4.1]) | p=0.020 |
| EQ-5D-5L VAS | | 67.9 (17.6) [n=434] | 63.7 (18.2) [n=259] | 4.2 (95% CI [1.5, 7.0]) | p=0.003 |
| EQ-5D-5L Index | | 0.77 (0.18) [n=434] | 0.72 (0.21) [n=259] | 0.05 (95% CI [0.02, 0.08]) | p=0.002 |
| eMPower + human support versus eMPower self-directed | | | | | |
| HADS Total | | 11.3 (6.2) [n=222] | 11.9 (6.7) [n=214] | 0.6 (95% CI [−0.7, 1.8]) | p=0.360 |
| HADS-Anxiety | | 6.7 (3.6) [n=222] | 7.2 (3.8) [n=214] | 0.5 (95% CI [−0.2, 1.2]) | p=0.160 |
| HADS-Depression | | 4.6 (3.4) [n=222] | 4.7 (3.7) [n=214] | 0.1 (95% CI [−0.6, 0.7]) | p=0.837 |
| MFIS Total | | 36.5 (15.8) [n=217] | 38.3 (16.6) [n=209] | 1.7 (95% CI [−1.3, 4.8]) | p=0.265 |
| MFIS Physical | | 17.8 (7.7) [n=217] | 18.7 (8.3) [n=209] | 0.9 (95% CI [−0.6, 2.4]) | p=0.254 |
| MFIS Cognitive | | 15.3 (7.8) [n=217] | 16.0 (8.0) [n=209] | 0.7 (95% CI [−0.8, 2.2]) | p=0.360 |
| MFIS Psychosocial | | 3.4 (2.0) [n=217] | 3.6 (2.0) [n=209] | 0.2 (95% CI [−0.2, 0.5]) | p=0.403 |
| SF-12 MCS | | 50.8 (7.7) [n=222] | 50.8 (8.3) [n=213] | 0.04 (95% CI [−1.5, 1.5]) | p=0.959 |
| SF-12 PCS | | 40.2 (11.9) [n=222] | 39.5 (12.4) [n=213] | −0.7 (95% CI [−3.0, 1.6]) | p=0.536 |
| EQ-5D-5L VAS | | 68.1 (17.7) [n=221] | 67.7 (17.5) [n=213] | −0.5 (95% CI [−3.8, 2.8]) | p=0.769 |
| EQ-5D-5L Index | | 0.78 (0.17) [n=221] | 0.77 (0.18) [n=213] | −0.01 (95% CI [−0.04, 0.02]) | p=0.559 |
| Sensitivity analysis (LOCF)  eMPower + human support versus control | | | | | |
| HADS Total | | 12.1 (6.7) [n=276] | 14.3 (7.1) [n=274] | 2.2 (95% CI [1.1, 3.4]) | p<0.001 |
| HADS-Anxiety | | 7.1 (3.9) [n=276] | 8.2 (4.2) [n=274] | 1.1 (95% CI [0.5, 1.8]) | p=0.001 |
| HADS-Depression | | 5.0 (3.7) [n=276] | 6.1 (3.7) [n=274] | 1.1 (95% CI [0.5, 1.7]) | p<0.001 |

Values are 12-week means (SD). Unadjusted mean differences and 95% CIs were derived from independent-samples t-tests without covariate adjustment. Welch-corrected t-tests were used where variances were unequal. Positive mean differences for HADS and MFIS indicate lower (better) scores in the intervention group; positive differences for SF-12 and EQ-5D indicate higher (better) scores. The LOCF sensitivity analysis imputed baseline values for participants with missing 12-week HADS data and was restricted to the primary comparison (eMPower + human support vs. control). Corresponding adjusted analyses are presented in Tables 3, S3, S4, and S5. HADS = Hospital Anxiety and Depression Scale; MFIS = Modified Fatigue Impact Scale; SF-12 = Short Form-12; MCS = Mental Component Summary; PCS = Physical Component Summary; VAS = Visual Analog Scale; EQ-5D-5L = EuroQol 5-Dimension 5-Level; LOCF = last observation carried forward; SD = standard deviation; CI = confidence interval.
