# Supplementary material for: Effect of a digital intervention on mental health symptoms in adults with chronic conditions: A three-arm randomized controlled trial
Source: PLoS Med. 2026 Aug 20;23(8):e1005198. doi: 10.1371/journal.pmed.1005198 (PMC13492819; doi:10.1371/journal.pmed.1005198)
Supplement: Supplementary file 10 [file pmed.1005198.s008.docx]

**Statistical Analysis Plan – Primary Paper**

Based on JAMA. 2017; 318(23):2337-2343. doi:10.1001/jama.2017.18556

**Trial:** eMPower: An online Mind-body wellness Program for adults living with chronic health conditions: A three-armed randomized controlled trial

**Trial Registration:** ClinicalTrials.gov Identifier: NCT05786482

**PI**: Puneeta Tandon

Protocol Number: 1

Protocol Date: 30 January 2025

Table of Contents

[Abbreviations	4](#_Toc228372370)

[Section 1: Administrative Information	5](#_Toc228372371)

[1.1 SAP revisions	5](#_Toc228372372)

[1.2 Roles and responsibility	5](#_Toc228372373)

[1.3 Signatures	5](#_Toc228372374)

[Section 2: Introduction	6](#_Toc228372375)

[2.1 Background and rationale	6](#_Toc228372376)

[2.2 Objectives	6](#_Toc228372377)

[Section 3: Study Methods	7](#_Toc228372378)

[3.1 Trial design	7](#_Toc228372379)

[3.2 Brief description of the interventions	7](#_Toc228372380)

[3.3 Randomization	8](#_Toc228372381)

[3.4 Sample size	8](#_Toc228372382)

[3.5 Framework	9](#_Toc228372383)

[3.6 Statistical interim analyses and stopping guidance	9](#_Toc228372384)

[3.7 Timing of final analysis	9](#_Toc228372385)

[3.8 Timing of outcome assessments	9](#_Toc228372386)

[Section 4: Statistical Principles	9](#_Toc228372387)

[4.1 Confidence intervals and P values	9](#_Toc228372388)

[4.2 Adherence and protocol deviations	9](#_Toc228372389)

[4.3 Analysis populations	9](#_Toc228372390)

[The analysis is based on the intention-to-treat (ITT) principle. This means that all randomized patients will be included in the analysis according to randomized treatment assignment.	9](#_Toc228372391)

[Section 5: Trial Population	9](#_Toc228372392)

[5.1 Screening data	9](#_Toc228372393)

[5.2 Eligibility	10](#_Toc228372394)

[5.3 Recruitment	10](#_Toc228372395)

[5.4 Withdrawal/follow-up	10](#_Toc228372396)

[5.5 Baseline patient characteristics	10](#_Toc228372397)

[6: Analysis	10](#_Toc228372398)

[6.1 Outcome definitions List	10](#_Toc228372399)

[6.2 Analysis methods	11](#_Toc228372400)

[6.2.1 Primary outcome	11](#_Toc228372401)

[6.2.2 Secondary outcome	11](#_Toc228372402)

[6.2.3 Covariates	11](#_Toc228372403)

[6.3 Missing data	11](#_Toc228372404)

[6.4 Statistical software	11](#_Toc228372405)

# Abbreviations

| AE | Adverse Event |
| --- | --- |
| ANCOVA | Analysis of Covariance |
| HADS | Hospital Anxiety and Depression Scale |
| WLC | Waitlist Control |
| SF-12 | Short Form-12 |
| KDQoL | Kidney Disease Quality of Life |
| CLDQ | Chronic Liver Disease Questionnaire |
| KCCQ-12 | Kansas City Cardiomyopathy Questionnaire |
| SAQ | Seattle Angina Questionnaire |
| PBC-40 | Primary Biliary Cholangitis-40 |
| SIBDQoL | Short Inflammatory Bowel Disease Quality of Life Questionnaire |
| EORTC – QLQ – C30 | The EORTC Core Quality of Life Questionnaire |
| MFIS | Modified Fatigue Impact Scale |
| SF-8 | Short Form 8 |
| COM-B | Capability, Opportunity, Motivation – Behaviour |
| USE | User Satisfaction, Ease of Use Scale |
| REDCap | Research Electronic Data Capture |
| CONSORT | Consolidated Standards of Reporting Trials |
| RCT | Randomized Controlled Trial |
| SD | Standard Deviation |
| ITT | Intention to treat |
| PBC | Primary Biliary Cholangitis |
| IBD | Inflammatory Bowel Disease |
| HF | Heart Failure |
| CKD | Chronic Kidney Disease |
| PT | Post Transplant |
| PTSD | Post Traumatic Stress Disorder |
| IQR | Interquartile Range |
| CI | Confidence Interval |
| MAR | Missing at Random |
| PROMIS | Patient Reported Outcomes Measurement Information System |
| SAS | Statistical Analysis System |
| PHN | Personal Healthcare Number |

# Section 1: Administrative Information

## 1.1 SAP revisions

| Protocol  version | Updated  SAP version no. | Section number  changed | Description of and reason for change | Date  changed |
| --- | --- | --- | --- | --- |
| 1 |  |  |  |  |
| 1 | 2.0 |  | Adapted to focus on primary and secondary outcomes and include prospective tables | 25 November 2024 |
| 1 | 3.0 |  | Finalized tables | 30 January 2025 |

## 1.2 Roles and responsibility

5 Names, affiliations, and roles of SAP contributors

| **Contributor** | **Affiliation** | **Role of Contributor** |
| --- | --- | --- |
| Puneeta Tandon | University of Alberta | Study PI |
| Yinggan Zheng | University of Alberta |  |
| Emily Johnson | University of Alberta |  |

## 1.3 Signatures

**Authors of eMPower Study SAP:**

| **Name (print):** | Emily Johnson |
| --- | --- |
| **Signature:** | EJ |
| **Date (dd-mmm-yyyy):** | 22 April 2024 |

| **Name (print):** | Yinggan Zheng |
| --- | --- |
| **Signature:** | YZ |
| **Date (dd-mmm-yyyy):** | 22 April 2024 |

**Principal Investigator of EMPOWER:**

| **Name (print):** | Dr. Puneeta Tandon |
| --- | --- |
| **Signature:** | PT |
| **Date (dd-mmm-yyyy):** | 22 April 2024 |

# **Section 2: Introduction**

## 2.1 Background and rationale

Chronic health conditions are a major global challenge, causing high rates of disability and reducing quality of life. Mind-body wellness practices, such as meditation, yoga, and cognitive behavioral therapies like Acceptance and Commitment Therapy (ACT), show promise in symptom relief and well-being. However, more research is needed on mixed-condition interventions, combining ACT with mindful practices, and enhancing online engagement. To address these needs, the eMPower RCT will evaluate an online mind-body program for people with chronic conditions. Building on previous trials, this study will test ACT combined with mindful practices and explore the impact of varied levels of support on outcomes.

The eMPower trial will assess an intervention that combines ACT with other mind-body techniques for people with chronic health conditions. It will compare two delivery formats: one with brief weekly check-ins and one without. The main outcome is to assess the impact of brief check-ins on anxiety and depression compared to a waitlist control group accounting for baseline HADS-total score. Secondary outcomes include fatigue and quality of life accounting for baseline scores.

## 2.2 Objectives

**Table 1. Objectives and related endpoints**

|  | **Objective** | **Endpoint** | **Measurement time points** |
| --- | --- | --- | --- |
| **Primary outcome** | Assessment of **depressive and anxiety symptoms** after intervention | Hospital Anxiety and Depression Scale (HADS) | Baseline (week 0), Week 12 |
| **Secondary outcomes** | Assessment of **quality of life** after intervention | Short Form-12 (SF-12) | Baseline (week 0), Week 12 |
|  | Assessment of **fatigue** after the intervention | Modified Fatigue Impact Scale (MFIS) | Baseline (week 0), Week 12 |
|  | Assessment of **Anxiety** after the intervention | HADS-Anxiety subscale | Baseline (week 0), Week 12 |
|  | Assessment of **Depression** after the intervention | HADS-Depression subscale | Baseline (week 0), Week 12 |

***2.2.1 Primary objective***

The sample size was powered for the single prespecified primary comparison: eMPower + human support (study arm 3) versus control (study arm 1) on HADS total score at 12 weeks. Additional sample size calculations were completed to determine the sample size required to detect a difference in the eMPower + weekly check-ins and waitlist controls if one exists.

The primary hypothesis is:

1. eMPower + 1-to-1 weekly check-ins (study arm 3) is more effective than control conditions in reducing symptoms of anxiety and depression in adults across chronic physical conditions.

***2.2.2 Secondary objective(s)***

All other pairwise comparisons (self-directed eMPower vs control; eMPower + human support vs self-directed eMPower; combined intervention arms vs control) are exploratory, not powered, and interpreted without confirmatory inference. No formal multiplicity adjustment will be applied to these exploratory comparisons. ANCOVA models will be adjusted for baseline score, chronic condition type, age, and sex.

# **Section 3: Study Methods**

## 3.1 Trial design

eMPower is a three-armed, pragmatic, randomized, controlled trial. Participants are randomized to one of three groups after informed consent and a 30-minute orientation with our study team. One group uses only eMPower for 12 weeks, the other group uses eMPower with an added brief (<15-minute) 1-to-1 weekly check-in for 12 weeks and the third group is a waitlist control group.

## 3.2 Brief description of the interventions

*Control group (Waitlist control, study arm 1)*

The control group will receive standard care. All control group participants will be offered access to the program following their waitlist period.

*eMPower 12-week online wellness program (study arm 2)*

The eMPower intervention comprises 12-weeks of recorded video content, including 5 levels of guided mindful movement routines at differing lengths (warm-up, tai-chi, yoga, chair movement, meditation, breathwork), an acceptance and commitment therapy-based coping skills program, and education from clinicians in their specific condition. Participants also have access to weekly group movement sessions.

*eMPower 12-week online wellness program + weekly ≤15-minute check-ins (study arm 3)*

The eMPower intervention comprises 12-weeks of recorded video content, including 5 levels of guided mindful movement routines at differing lengths (warm-up, tai-chi, yoga, chair movement, meditation, breathwork), psychology content, and education from clinicians in their specific condition. Participants also have access to weekly group movement sessions. In addition, participants in this arm will receive a weekly ≤15-minute phone call from a member of the research team.

## 3.3 Randomization

Randomization was conducted electronically in REDCap using computer-generated permuted blocks of size 6 within eight predefined chronic medical condition strata in a 1:1:1 allocation ratio. Study arms are balanced according to chronic physical condition type. The randomization is performed via an allocation table created by a statistician and administered through the electronic database REDCap^1^. To ensure concealed allocation, eligibility is determined, and all baseline surveys completed before randomization in compliance with CONSORT guidelines (CONSORT 2025, CONSORT 2010+extension)^2^.

## 3.4 Sample size

The sample size for the HADS total primary outcome was conducted using G*power (Version 3.1.9.7)^3^. The individual data from two previous RCTs carried out in single condition group populations of IBD and PBC compared an intervention similar to eMPower study arm 3 against waitlist controls with standardized effect sizes of 0.8 and 0.5, respectively^4,5^. Assuming an effect size of 0.2 in arm 2 (eMPower) and 0.5 in arm 3 (eMPower + brief 1-to-1 weekly check-ins), with an alpha of 0.05 and 80% power, a sample of 309 participants (103 per arm) will be adequate to detect a statistically significant mean difference ^6^ in the HADS total primary outcome (**Table 2)**. A sample size calculation was also carried out to determine how many participants will be required to detect a statistically significant mean difference between the two intervention arms (arm 2 (eMPower) and arm 3 (eMPower + brief 1-to-1 weekly check-ins)). Assuming an effect size of 0.2 in arm 2 and 0.5 in arm 3, this will require a sample size of 864 participants (288 per arm). Within the time and funding limitations of the grant, we will enroll at least 309 participants up to a maximum of 864 participants.

**Table 2.** Sample size contingency table

| Arm 1 (waitlist controls) | Arm 2 (eMPower) | Arm 3 (eMPower + check-ins) | Comparison tested | Total Sample Size | Total Sample Size + 20% drop out |
| --- | --- | --- | --- | --- | --- |
| 0 | 0.2 | 0.4 | Omnibus | 366 | 459 |
|  |  |  | Arm 1 vs Arm 2 | 1524 | 1905 |
|  |  |  | Arm 2 vs Arm 3 | 1527 | 1920 |
|  |  |  | Arm 3 vs Arm 1 | 378 | 474 |
| 0 | 0.2 | 0.5 | Omnibus | 231 | 291 |
|  |  |  | Arm 1 vs Arm 2 | 1524 | 1905 |
|  |  |  | Arm 2 vs Arm 3 | 690 | 864 |
|  |  |  | Arm 3 vs Arm 1 | 246 | 309 |

*Adapted from Brooks and Johanson (2011)* ^6^

## 3.5 Framework

The study follows the CONSORT 2025 statement and CONSORT 2010 + extension for multi-arm trials^7,8^, relevant sections of the SPIRIT guidelines^9^, and the UK Medical Research Council’s framework for complex interventions^10^. It is registered on <https://clinicaltrials.gov/> (Trial Registration: NCT05786482, registered on March 9, 2023) and has received approval from the Research Ethics Office at the University of Alberta (Pro00122568).

## 3.6 Statistical interim analyses and stopping guidance

No interim analyses or guidelines for stopping the trial early are planned. There are no predefined stopping rules.

## 3.7 Timing of final analysis

The final analysis of the primary/secondary outcomes will be performed when the last participant has completed their 12-week waitlist or intervention period.

## 3.8 Timing of outcome assessments

The primary outcome is measured at baseline (0 weeks) and end of intervention/waitlist period (12 weeks).

# **Section 4: Statistical Principles**

## 4.1 Confidence intervals and P values

Analyses followed a prespecified hierarchical testing strategy. An omnibus ANCOVA first assessed overall differences across the three randomized arms at α=0.05. If the omnibus test was statistically significant, the single prespecified primary pairwise comparison (eMPower + human support vs control) was examined at α=0.05. This hierarchical gatekeeping approach controls the Type I error rate for the primary comparison.

## 4.2 Adherence and protocol deviations

All trial data is logged in individual forms in REDCap. Reasons for withdrawal from the trial such as health-related complications and death are recorded.

## 4.3 Analysis populations

## The analysis is based on the intention-to-treat (ITT) principle. This means that all randomized patients will be included in the analysis according to randomized treatment assignment.

# **Section 5: Trial Population**

## 5.1 Screening data

Screening takes place online via REDCap. Available data on participants assessed for eligibility will be included within a CONSORT flow diagram.

## 5.2 Eligibility

*Inclusion criteria:*

- 18 years and older
- Live with a chronic physical condition, with specific programming for: Primary Biliary Cholangitis (PBC), individuals who are post-transplant, cirrhosis, chronic digestive disease, Heart Failure (HF), chronic kidney disease (CKD), primary sclerosing cholangitis (PSC) or have another chronic physical condition (included in a general group)
- Ability to communicate in English
- Ability to access to an Internet-connected device in their home

*Exclusion criteria:*

- Participants who fail to sign informed consent
- Live with severe psychiatric disorders
  - Uncontrolled post-traumatic stress disorder (PTSD) unless approved to participate by their physician/psychologist
  - Uncontrolled bipolar disorder, unless approved to participate by their physician/psychologist
  - Uncontrolled schizophrenia, unless approved to participate by their physician/psychologist
  - Suicidality nearly every day

## 5.3 Recruitment

The CONSORT flow diagram will show patient characteristics including sample sizes for recruitment, randomization, and analyses.

## 5.4 Withdrawal/follow-up

If a randomized participant withdraws from the intervention, we will offer to complete some or all the end-of-study survey items. The number/frequency of participants lost to follow-up will be provided for each study arm. If possible, the reasons for participants not completing the trial will be given (e.g., lost to follow up, illness, time limitations, etc.).

## 5.5 Baseline patient characteristics

Discrete variables will be summarised as frequencies and percentages. Continuous variables will be summarised by the mean and standard deviation (SD) or by the median and interquartile range (IQR). Durations and time intervals will be summarised by medians and IQRs.

# **6: Analysis**

## 6.1 Outcome definitions List

**Primary outcome**

The primary outcome was the Hospital Anxiety and Depression Scale (HADS) total score at 12 weeks, analyzed using ANCOVA adjusting for baseline HADS total score, age, sex, and chronic condition^11^.

**Secondary outcome(s)**

*Quality of life* is measured in all participants by the Short Form 12 (SF-12)^12^ and by a chronic physical condition-specific measure when available, from baseline (0 weeks) to end-of-intervention/control period (12 weeks). Health-related quality of life is also assessed using EQ-5D-5L index scores calculated with country-specific value sets.

*Fatigue* is measured at baseline and at week 12 with the MFIS^13^.

## 6.2 Analysis methods

### 6.2.1 Primary outcome

To analyze the impact of the intervention on the HADS total, we will use analysis of covariance (ANCOVA). Model assumptions (normality and homoscedasticity) will be assessed using residual diagnostics; transformations were applied as needed. Log transformation will be applied as needed. The ANCOVA model will contain baseline HADS total score, intervention group, chronic condition type, age, and sex as covariates. The beta coefficient of the intervention variable will represent the absolute improvement of the intervention and relative improvement of the intervention will be defined as the percentage difference in HADS total score at the end of the study between the intervention and control group (absolute improvement ÷ mean end of study HADS total score in control group score at 12 weeks). Secondary outcomes were analyzed using ANCOVA models adjusting for baseline score, chronic condition type, age, and sex (see **Tables 2, 3 and 4**). All statistical tests will be two-sided with an alpha=0.05 unless otherwise specified.

### 6.2.2 Secondary outcome

The analyses of secondary endpoints quality of life (SF-12), fatigue (MFIS), and anxiety and depression subscales (HADS-A/HADS-D) will follow the similar approach as the analyses described for the primary endpoint.

### 6.2.3 Covariates

All ANCOVA models were adjusted for baseline score, chronic condition type, age, and sex, given their known associations with digital intervention engagement and mental health outcomes.

## 6.3 Missing data

The primary analysis will be reported using available cases without imputation. A sensitivity analysis using last observation carried forward (LOCF) will be conducted for the primary comparison (eMPower + human support vs control) on HADS total to assess robustness to missing data assumptions.

## 6.4 Statistical software

The statistical analysis will be performed using SPSS (Version 24.0; IBM Corp., Armonk, NY).

**References**

1.	Harris P, Taylor R, Minor B, et al. The REDCap consortium: Building an international comunity of software partners. J Biomed 2019. DOI: doi: 10.1016/j.jbi.2019.103208.

2.	Rennie D. CONSORT revised--improving the reporting of randomized trials. Jama 2001;285(15):2006–7. (In eng). DOI: 10.1001/jama.285.15.2006.

3.	Faul F, Erdfelder E, Lang A-G, Buchner A. G*Power 3: A flexible statistical power analysis program for the social, behavioral, and biomedical sciences. Behavior Research Methods 2007;39(2):175–191. DOI: 10.3758/BF03193146.

4.	Peerani F, Watt M, Ismond KP, et al. A randomized controlled trial of a multicomponent online stress reduction intervention in inflammatory bowel disease. Therap Adv Gastroenterol 2022;15:17562848221127238. (In eng). DOI: 10.1177/17562848221127238.

5.	Watt M, Hyde A, Johnson E, et al. An online mind-body program improves mental health and quality of life in primary biliary cholangitis: A randomized controlled trial. Hepatology Communications 2023;7(11):e0316. DOI: 10.1097/hc9.0000000000000316.

6.	Brooks G, Johanson G. Sample Size Considerations for Multiple Comparison Procedures in ANOVA. Journal of Modern Applied Statistical Methods 2011;10:97–100. DOI: 10.22237/jmasm/1304222940.

7.	Juszczak E, Altman DG, Hopewell S, Schulz K. Reporting of Multi-Arm Parallel-Group Randomized Trials: Extension of the CONSORT 2010 Statement. JAMA 2019;321(16):1610–1620. DOI: 10.1001/jama.2019.3087.

8.	Hopewell S, Chan AW, Collins GS, et al. CONSORT 2025 statement: updated guideline for reporting randomized trials. Nat Med 2025;31(6):1776–1783. (In eng). DOI: 10.1038/s41591-025-03635-5.

9.	Chan AW, Tetzlaff JM, Gøtzsche PC, et al. SPIRIT 2013 explanation and elaboration: guidance for protocols of clinical trials. BMJ 2013;346:e7586. (In eng). DOI: 10.1136/bmj.e7586.

10.	Kathryn S, Lynsay M, Sharon Anne S, et al. A new framework for developing and evaluating complex interventions: update of Medical Research Council guidance. BMJ 2021;374:n2061. DOI: 10.1136/bmj.n2061.

11.	Zigmond AS, Snaith RP. The hospital anxiety and depression scale. Acta Psychiatr Scand 1983;67(6):361–70. (In eng). DOI: 10.1111/j.1600-0447.1983.tb09716.x.

12.	Turner-Bowker D, Hogue SJ. Short Form 12 Health Survey (SF-12). Springer Netherlands; 2014:5954–5957.

13.	Larson RD. Psychometric properties of the modified fatigue impact scale. Int J MS Care 2013;15(1):15–20. (In eng). DOI: 10.7224/1537-2073.2012-019.
