# Supplementary material for: Effect of a digital intervention on mental health symptoms in adults with chronic conditions: A three-arm randomized controlled trial
Source: PLoS Med. 2026 Aug 20;23(8):e1005198. doi: 10.1371/journal.pmed.1005198 (PMC13492819; doi:10.1371/journal.pmed.1005198)
Supplement: Supplementary file 11 [file pmed.1005198.s009.docx]

**S7 Table. Multiplicity adjustment for secondary outcomes using Hochberg and Bonferroni corrections: eMPower + human support versus control at 12 weeks (intention-to-treat population)**

| **Hochberg rank (k)** | **Secondary outcome** | **Adjusted mean difference (95% CI)** | **Between-group relative improvement (%) (95% CI)** | **F(df), p-value, partial ƞ²** | **Hochberg Threshold (α/k)** | **Significant after Hochberg?** | **Significant after Bonferroni (α/6 = 0.0083)?** |
| --- | --- | --- | --- | --- | --- | --- | --- |
| 1 | SF-12 PCS | 1.9 (95% CI [0.5, 3.4]) | 5.1% (95% CI [1.3, 9.0]) | F(1,475)=7.27; p=0.009; partial ƞ²=0.02 | 0.0500 | Yes | No |
| 2 | EQ-5D-5L Index Score | 0.05 (95% CI [0.03, 0.08]) | 7.1% (95% CI [4.3, 11.4]) | F(1,474)=18.7; p<0.001; partial ƞ²=0.04 | 0.0250 | Yes | Yes |
| 3 | SF-12 MCS | 3.0 (95% CI [1.8, 4.2]) | 6.3% (95% CI [3.8, 8.8]) | F(1,475)=25.2; p<0.001; partial ƞ²=0.05 | 0.0167 | Yes | Yes |
| 4 | HADS-Depression | 1.4 (95% CI [0.9, 1.9]) | 23.0% (95% CI [14.8, 31.1]) | F(1,475)=28.0; p<0.001; partial ƞ²=0.06 | 0.0125 | Yes | Yes |
| 5 | HADS-Anxiety | 1.5 (95% CI [1.0, 2.0]) | 18.3% (95% CI [12.2, 24.4]) | F(1,475)=36.9; p<0.001; partial ƞ²=0.07 | 0.0100 | Yes | Yes |
| 6 | MFIS total | 8.4 (95% CI [6.6, 10.4]) | 18.8% (95% CI [14.8, 23.3]) | F(1,469)=78.9; p<0.001; partial ƞ²=0.10 | 0.0083 | Yes | Yes |

Multiplicity corrections were applied across six prespecified secondary outcomes for the primary comparison (eMPower + human support vs control) to control the familywise Type I error rate. The Hochberg step-up procedure was used as the primary method; Bonferroni correction (α = 0.0083) was applied as a conservative sensitivity analysis. Adjusted mean differences were estimated using ANCOVA adjusting for baseline score, chronic condition type, age, and sex. Full descriptive statistics are presented in Table 3. Higher HADS and MFIS scores indicate greater symptom burden; higher SF-12 and EQ-5D-5L scores indicate better quality of life. Positive adjusted differences favor the intervention in all cases. CI = confidence interval; HADS = Hospital Anxiety and Depression Scale; MFIS = Modified Fatigue Impact Scale; SF-12 = Short Form-12; MCS = Mental Component Summary; PCS = Physical Component Summary; VAS = Visual Analog Scale; SD = standard deviation; df = degrees of freedom.
