# Supplementary material for: Effect of a digital intervention on mental health symptoms in adults with chronic conditions: A three-arm randomized controlled trial
Source: PLoS Med. 2026 Aug 20;23(8):e1005198. doi: 10.1371/journal.pmed.1005198 (PMC13492819; doi:10.1371/journal.pmed.1005198)
Supplement: Supplementary file 12 [file pmed.1005198.s010.docx]

**Semi-structured script for weekly check-ins**

**Opening**
"Hello [participant name], this is [research staff name] calling for your weekly eMPower check-in. Do you have a few minutes to go through some questions about how this past week went with the program?"
*Record: Check-in date (D-M-Y) and participant week number.*

**Program adherence**
"I'd like to ask you about how often you engaged with each part of the program this past week."
"About how many days this week did you complete the **Life Skills** portion of the program?"
*(Record number of days)*

"About how many days this week did you complete the **Energize** portion of the program?"
*(Record number of days)*

**Weekly experience**
"How did this week go for you?”
*(Record open-ended response)*

**Participant experience**
"What did you like best about the program this week?"
*(Record open-ended response)*

"What issues did you encounter this week — for example, with technology — and what suggestions would you make to improve this week's program?"
*(Record open-ended response)*

**Additional details and closing**
*Record any additional details, observations, or notable comments from the check-in.*

"Thank you for taking the time to check in this week. We really appreciate your participation. Do you have any other questions or concerns before we wrap up?"

*Confirm the date/time for the check-in next week.*
