# Supplementary material for: Effect of a digital intervention on mental health symptoms in adults with chronic conditions: A three-arm randomized controlled trial
Source: PLoS Med. 2026 Aug 20;23(8):e1005198. doi: 10.1371/journal.pmed.1005198 (PMC13492819; doi:10.1371/journal.pmed.1005198)
Supplement: Supplementary file 13 [file pmed.1005198.s012.docx]

**Title:** eMPower: An online Mind-body wellness Program for adults living with chronic health conditions: A three-armed randomized controlled trial protocol

**Authors:**

Emily Johnson, MSc

Faculty of Medicine and Dentistry

Division of Gastroenterology (Liver Unit), University of Alberta, Edmonton, Alberta

Email: [ecjohnso@ualberta.ca](mailto:ecjohnso@ualberta.ca)

Kathleen Ismond, MLIS, MSc, PhD

Faculty of Medicine and Dentistry

Division of Gastroenterology (Liver Unit), University of Alberta, Edmonton, Alberta

Email: [kismond@ualberta.ca](mailto:kismond@ualberta.ca)

Ashley Hyde, PhD

Faculty of Nursing, University of Alberta, Edmonton, Alberta

Email: [ac20@ualberta.ca](mailto:ac20@ualberta.ca)

Justin Ezekowitz, MBBCh, MSc

Faculty of Medicine and Dentistry

Division of Medicine, University of Alberta, Edmonton, Alberta

Email: [jae2@ualberta.ca](mailto:jae2@ualberta.ca)

Gail Wright

Canadian Primary Biliary Cholangitis (PBC) Society

Email: [gail.wright@pbc-socety.ca](mailto:gail.wright@pbc-socety.ca)

Jude Spiers, PhD

Faculty of Nursing, University of Alberta, Edmonton, Alberta

Email: [jaspiers@ualberta.ca](mailto:jaspiers@ualberta.ca)

John Spence, PhD

Faculty of Kinesiology, Sport, and Recreation

University of Alberta, Edmonton, Alberta

Email: jc.spence@ualberta.ca

Stephanie Thompson, MD, MSc

Faculty of Medicine and Dentistry

Division of Medicine, University of Alberta, Edmonton, Alberta

Email: [th11@ualberta.ca](mailto:th11@ualberta.ca)

Margaret L. McNeely, PhD

Faculty of Rehabilitation Medicine

University of Alberta, Edmonton, Alberta

Email: [mmcneely@ualberta.ca](mailto:mmcneely@ualberta.ca)

Yinggan Zheng, MA, MEd

Faculty of Medicine and Dentistry

Division of Medicine, University of Alberta, Edmonton, Alberta

Email: [yinggan@ualberta.ca](mailto:yinggan@ualberta.ca)

Jeff Round, MA, PhD

School of Public Health

University of Alberta, Edmonton, Alberta

Email: [jround@ualberta.ca](mailto:jround@ualberta.ca)

Sarah Tymchuk, MD

Faculty of Medicine and Dentistry

Department of Psychiatry University of Alberta, Edmonton, Alberta

Email: [tymchuk@ualberta.ca](mailto:tymchuk@ualberta.ca)

Dayna Lee-Baggley, PhD

Department of Family Medicine

Dalhousie University, Halifax, Nova Scotia

Email: dayna.lee-baggley@dal.ca

Serena Isley

Division of Gastroenterology (Liver Unit), University of Alberta, Edmonton, Alberta

Email: [isley@ualberta.ca](mailto:isley@ualberta.ca)

Shaina Corrick

Division of Gastroenterology (Liver Unit), University of Alberta, Edmonton, Alberta

Email: [corrick@ualberta.ca](mailto:corrick@ualberta.ca)

Holly Minckler

Division of Gastroenterology (Liver Unit), University of Alberta, Edmonton, Alberta

Email: [hminckle@ualberta.ca](mailto:hminckle@ualberta.ca)

Puneeta Tandon, MD, MSc Epidemiology

Division of Gastroenterology (Liver Unit), University of Alberta, Edmonton, Alberta

Email: [ptandon@ualberta.ca](mailto:ptandon@ualberta.ca)

**Corresponding Author:**

Dr. Puneeta Tandon, Division of Gastroenterology (Liver Unit), Department of Medicine, Division of Gastroenterology (Liver Unit), Edmonton, Alberta, Canada. Email: ptandon@ualberta.ca; fax: 7806424170

**Conflicts of Interest and Source of Funding:** None of the authors have any conflicts of interest to declare. This manuscript is funded through a grant by the Canadian Institutes for Health Research (CIHR) awarded to PT (Grant award number: #179974). Additional funding from TRIANGLE (TraIning A New generation of researchers in Gastroenterology and LivEr), Mitacs with Canadian PBC society and HeartLife to partially support salaries of EJ, SI, SC respectively.

**Ethics approval:** Approved 21 November 2022 by the University of Alberta Health Research Ethics Board (HREB Pro #00122568).

**Trial registration:** Registered at clinicaltrials.gov (Trial Registration: NCT05786482, Date of registration: March 27, 2023).

**Acknowledgements:** We graciously acknowledge the support of the following patient partner organizations: HeartLife, the Canadian Primary Biliary Cholangitis (PBC) Society, PBCers, The Kidney Foundation of Canada including the Northern Alberta and Territories Branch, the Canadian Liver Foundation, and the Canadian Digestive Health Foundation. We also acknowledge the support of our patient partners and clinicians who have contributed to content review and recruitment. Thank you also to the many clinical team members for who have provided content and provided guidance on recruitment.

**Number of tables: 2**

**Number of figures: 2**

**Abstract**

**Background:** Symptoms of depression and anxiety are prevalent among adults with chronic health conditions, contributing to reduced quality of life, morbidity, and mortality. Mind-body wellness interventions (i.e. psychology programming, mindful movement, breathwork, meditation) may impact mental health symptoms, with online delivery offering access and scalability. Whether online mind-body wellness interventions are effective in improving patient outcomes across a broad range of chronic conditions remains uncertain.

**Methods:** This three-armed, pragmatic, randomized controlled trial will use a nested mixed methods approach to assess the effectiveness of an online mind-body wellness intervention (eMPower), offered at two levels of personnel support, on symptoms of anxiety and depression in adults with chronic health conditions. Inclusion criteria require a self-reported chronic condition and access to an internet-connected device. Eligible participants will be randomized 1:1:1 to (1) waitlist control; (2) eMPower; (3) eMPower + weekly 1-to-1 check-in. The primary analysis will compare the Hospital and Anxiety Depression Scale (HADS) total score between eMPower + weekly 1-to-1 check-ins versus controls, with secondary and exploratory outcomes including HADS subscales, health-related quality of life, fatigue, program engagement, and frailty.

**Conclusion:** With online intervention delivery, a range of outcomes, mixed method evaluation, and automated intervention tracking, findings are anticipated to enhance our understanding of how individuals living with chronic health conditions engage with and are impacted by online mind-body wellness programming. Six hundred and fifty-six participants have been enrolled as of April 5, 2024, and 598 patients have completed 12-week follow-up.

**Keywords:** eHealth, depression, anxiety, primary biliary cholangitis, post-transplant, breathwork

1. **Introduction**

Chronic health conditions are defined by the World Health Organization as conditions “*requiring ongoing management and treatment over extended periods of time.”* They are the leading cause of death and disability across the world(1, 2), and in North America account for over 65 million lost Disability Adjusted Life Years(2). Individuals with chronic health conditions face a high symptom burden, social challenges, stress, and co-existing mental or physical health issues, leading to increased rates of depression, anxiety, and reduced quality of life(3).

Mind-body wellness techniques are increasingly acknowledged for their ability to alleviate physical and mental health symptoms, enhance quality of life, and improve self-efficacy in individuals with chronic health conditions(4-6). These techniques include meditation, yoga, and Tai chi(7), as well as psychology-based interventions such as cognitive behavioral therapy (CBT) or derivatives like acceptance and commitment therapy (ACT)(8-11). Recent systematic reviews have reported that these non-pharmaceutical interventions can significantly improve anxiety, depression, and quality of life(12-15).

The emergence of online options for the delivery of mind-body techniques (e.g., apps, websites) offers broader accessibility and on-demand access to content. However, areas for further exploration remain. A systematic review and mixed methods analysis of depression and anxiety apps highlighted the importance of incorporating evidence-based techniques, finding that non-evidence-based apps had lower user ratings and fewer users found them beneficial for mental health compared to apps with evidence-based techniques(16). Additionally, a meta-analysis of 56 randomized controlled trials (RCTs) evaluated online mind-body wellness interventions in chronic conditions (n=7691 participants) highlighting important gaps(17). This included the need for more research on the impact of an intervention delivered across mixed chronic conditions to enhance scalability; 96% (54/56) of the included studies evaluated single conditions. Second, while the majority of studies evaluated CBT or one of its derivatives alone, limited information was available on studies that combined CBT alongside mindful movement, breathwork and/or meditation(17), a strategy that has been well received by patients in our previous research(18, 19). Third, mixed methods evaluation was infrequent, used in only 25% of trials despite its utility in understanding patient experiences(20). Finally, patient engagement and high dropout was noted as a challenge with dropout rates averaging 41% and varied methods for assessing engagement(17). This aligns with a previously published systematic review of 93 mental health apps noting adherence rates below 5% within 30 days(21) and calls for further research on strategies to maintain engagement and reduce dropouts. This includes the evaluation of engagement strategies that are embedded within online platforms (e.g. gamification elements including leaderboards, points)(22-24) as well as potentially more effective, but more costly personnel requiring strategies (e.g. weekly 1-to-1 check-ins).

To date, our research group has published two RCTs using an online mind-body intervention that combines mindful movement, breathwork and meditation content alongside ACT psychology content(18, 19). Both studies were carried out in single disease populations (inflammatory bowel disease (IBD) and primary biliary cholangitis (PBC)(18, 19)), with brief weekly 1-to-1 check-ins by non-psychologist study personnel. Study results demonstrated high acceptability and study completion with beneficial effects on anxiety and depression scores. While effective, our research has not evaluated the impact of this online mind-body intervention in a mixed chronic health condition group nor explored its effectiveness in the absence of brief 1-to-1 weekly check-ins from study personnel, a delivery modality that may increase feasibility and sustainability. Building from our experience to date and the existing gaps in the literature, we propose the eMPower RCT.

1.1 **Objectives**

The eMPower trial will recruit a diverse range of individuals with chronic health conditions to evaluate an intervention that combines ACT and other mind-body techniques delivered across two levels of personnel support: eMPower + brief 1-to1 weekly check-ins versus eMPower with no check-ins. In comparison to the waitlist control arm, the objectives are to determine:

1. The effects of the eMPower program with brief weekly 1-to-1 check-ins on the primary outcome of anxiety and depression symptoms.
2. The effects of the eMPower program with brief weekly 1-to-1 check-ins on secondary (e.g., fatigue, quality of life) and exploratory outcomes (e.g. sleep quality, frailty).
3. If a difference exists in outcomes between the two intervention arms.
2. **Methods**
   2.1 *Design*

This online three-armed RCT will use a nested mixed methods design and commenced in March 2023, with enrollment expected to conclude by Summer 2024. The study follows the CONSORT 2010 statement for multi-arm trials(25), relevant sections of the SPIRIT guidelines (**Appendix 1**)(26), and the UK Medical Research Council’s framework for complex interventions(27). It is registered on <https://clinicaltrials.gov/> (Trial Registration: NCT05786482, registered on March 27, 2023) and has received approval from the Research Ethics Office at the University of Alberta (Pro00122568).

2.2 *Participants and eligibility criteria*

Recruitment, intervention delivery, and data collection are conducted entirely online. Potential participants will be identified through patient partner organizations (see Acknowledgements), associated email lists, patient conferences, and social media. Clinical centres will also be informed about the study and provided with advertisement materials that direct interested individuals to the recruitment website for more information and registration. As part of registration, a consent form will be completed followed by a series of questionnaires hosted on REDCap(28).

*Inclusion criteria*: To be eligible for randomization, participants will have access to an internet-connected device, communicate in English, provide online consent, and self-report as living with a chronic health condition endorsed by our patient partner organizations PBC, cirrhosis, chronic kidney disease [CKD], post-organ transplant, heart failure, inflammatory bowel disease [IBD], chronic digestive disease(s), women who have had a cardiac event, or self-report as living with another chronic physical health condition). Additional chronic health conditions may be added during the trial period. There will be no enrollment restrictions based on medical, psychological, or psychiatric treatment. *Exclusion criteria*: Self-report as living with uncontrolled post-traumatic stress disorder, schizophrenia, or bipolar disorder, or experiencing suicidality ‘nearly every day,’ unless a letter of exception is obtained from a psychologist or physician.

During the recruitment period, the study’s steering committee approved two changes to the inclusion and exclusion criteria based on feedback from participants and patient partner organizations in order to increase inclusivity. On May 19th, 2023, with 180 participants enrolled, the age for inclusion was expanded from ≥ 50 years to ≥18 years. On October 21st, 2023, with 321 participants enrolled, the exclusion criteria of HADS-Depression subscale score >10 was removed. By that time, 72 participants had been excluded due to the HADS criteria. The trial steering committee, psychology/psychiatry team members and patient partner organizations were in agreement that safety could be maintained by retaining exclusion for self-reported suicidality ‘nearly every day’, and self-reported uncontrolled psychiatric symptoms alongside links within the program to access public mental health resources if required.

2.3 *Participant flow*

After completing baseline questionnaires, participants will attend a videoconference orientation session (30-minutes). Each session will occur in small groups led by a research team member who will introduce mind-body wellness programming, explain randomization and timelines depending on which arm the participant is randomized to, and review the features of the eMPower digital platform. Randomization will occur immediately after the orientation. The eMPower statistician (YZ) has created and validated allocation tables for upload to REDCap. These lists will remain concealed until random assignment. Participants will be randomly allocated 1:1:1 to three study arms (**Figure 1**), stratified by their chronic health condition:

Arm 1 – ‘*Waitlist Control’* participants will receive a weekly newsletter including a motivational wellness quote(18, 19).

Arm 2 – ‘*eMPower’* participants will receive access to the 12-week online mind-body wellness program, weekly newsletters highlighting program content, and optional weekly online group movement sessions (~45 minutes).

Arm 3 – ‘*eMPower + 1:1 weekly check-ins’* participants will receive all interventions from Arm 2 and have a brief (<15 minutes) 1-to-1 weekly telephone check-in with graduate students from the research team. All students undergo 2 days of motivational interviewing training and receive as needed support from the clinical psychologist (DLB). Check-ins follow a semi-structured outline intended to review the week’s programming experience and engagement, assess weekly goals, and troubleshoot potential technical challenges.

**Figure 1**. eMPower participant flow

2.4 *Intervention*

eMPower builds on our previous studies(18, 19, 29) with input from physicians, allied health professionals, technology experts, and use of the Capability, Opportunity, Motivation and Behaviour (COM-B) model to promote engagement. The COM-B model is a behavior change framework that provides a theoretical basis for understanding why behavior change may or may not have occurred(30). The online program will be accessed on a password-protected digital platform (www.empower.wellnesstoolbox.ca). Personally identifying data are limited to full name and email address. The data will be hosted in a centre located in Canada in compliance with General Data Protection Regulation (GDPR) requirements(31). eMPower will be comprised of two main components: (1) Energize and (2) Life Skills:

*2.4.1 Energize:*

This component offers breathwork, meditation and mindful movement, with options including five video-based mindful movement streams (yoga at two levels of intensity, tai chi, and chair exercises at two levels of intensity). The core breathwork techniques, drawing from our previous work, include video content demonstrating five practices that progress weekly: Shining Skull breath (not included for individuals with cardiac conditions), Diaphragmatic breathing, Sighs, Bee breath, and Breath awareness. The meditations, ranging from 4 to 17 minutes, include five sleep meditations(19, 32) (see **Appendix 2)**. While there is no strict adherence target provided, participants are informed during the orientation that 90 minutes per week of Energize activities have been associated with increased mental health and fatigue-related benefits in our previous research(19). Participants can choose between long routines (~35 minutes; combination of a warm-up, mindful movement, breathwork, meditation), short routines (~20 minutes; combination of a warm-up, meditation), and short video clips (~10 minutes; warm-up only, mindful movement only, breathwork only, meditation only) to suit various schedules and preferences.

2.4 *Life Skills: Coping skills program + Weekly Tip from your care team*

This program component is based on ACT(33), involving weekly videos and activities (e.g., values, sleep, and pacing) drawn from previous programming(19) (see **Appendix 3** for a list of topics). Additionally, participants receive weekly 5–15-minute ‘Tip from your care team’ videos from a clinician; some videos are shared across chronic health conditions and relate to living with a chronic health condition while other videos are specific to each chronic health condition group) (see **Appendix 4** for a list of topics).

2.5 *Features to promote engagement*

Gamified elements include weekly polls, points, badges, a leaderboard, and a weekly e-gift card raffle. Social features enable participants to give “likes” to others.

2.6 *Outcomes*

The primary outcome, symptoms of anxiety and depression, will be evaluated using the HADS total score at 12 weeks versus baseline. The HADS is a 14-item self-report tool validated for use in non-psychiatric medical outpatient settings(34). It was chosen for its ability to differentiate anxiety and depression symptoms and minimize overlap from disease-related somatic symptoms(34). Secondary outcomes include the HADS anxiety and HADS depression subscale scores, fatigue assessed using the Modified Fatigue Impact Scale (MFIS)(35), and quality of life assessed using the Short-Form 12 (SF-12)(36) , as well as a condition-specific measure when available:

- For chronic kidney disease, the Kidney Disease Quality of Life survey (KDQoL)(37)
- For cirrhosis, the Chronic Liver Disease Questionnaire (CLDQ)(38)
- For heart failure, the Kansas City Cardiomyopathy Questionnaire (KCCQ-12)(39)
- For women who have experienced a cardiac event, the Seattle Angina Questionnaire (SAQ)(40)
- For inflammatory bowel disease, the Short Inflammatory Bowel Disease Quality of Life Questionnaire (SIBDQoL)(41)
- For chronic conditions without a condition-specific measure, the Short Form Survey 36 (SF-36)(42)

Additional exploratory outcomes will be assessed as a change from baseline to 12 weeks, including frailty, sleep disturbance, demoralization, technology proficiency, program engagement, satisfaction, and program ease of use. Questionnaires (**Table 1**) will be completed by arms 2 and 3 at baseline, end-of-study, and 8-week follow-up (0, 12, 20 weeks). The waitlist control arm (arm 1) will complete these questionnaires at baseline and the end of waitlist control period (0, 12 weeks). If waitlist control participants opt into the intervention, their end-of-intervention data will be collected 24 weeks from study entry. A short survey based on the COM-B(43) will be sent to all intervention participants (weeks 4, 8 for arms 2 and 3 and weeks 16, 20 for arm 1). All assessments will be in English and accessed online via REDcap(28). Participants will receive up to three email/phone reminders to complete their questionnaires.

**Table 1. Overview of questionnaire measures**

| **Questionnaire measures** | **Baseline** (week 0) | **Week 4**  (arms 2 & 3 only) | **Week 8** (arms 2 & 3 only) | **Week 12** | **Week 16** (waitlist control arm only) | **Week 20** | **Week 24** (waitlist control arm only) |
| --- | --- | --- | --- | --- | --- | --- | --- |
| Sociodemographic questions | X |  |  |  |  |  |  |
| Anxiety and depression (Hospital Anxiety and Depression Scale [HADS])(34) | X |  |  | X |  | X | X |
| Quality of Life Scale: Short-Form 12 (with the addition of the remaining questions form the Short-Form 36 OR a condition-specific measure when available)(36-42, 44) | X |  |  | X |  | X | X |
| Fatigue (Modified Fatigue Impact Scale [MFIS])(35) | X |  |  | X |  | X | X |
| Frailty (Fried Frailty Score(45), Edmonton Frail Scale [EFS](46)) | X |  |  | X |  | X | X |
| Sleep disturbance (Short Form-8)(47) | X |  |  | X |  | X | X |
| Demoralization (Demoralization-II)(48) | X |  |  | X |  | X | X |
| EQ5D-5L(49) | X |  |  | X |  | X | X |
| Technological proficiency(50) | X |  |  | X |  | X | X |
| COM-B behavioral factors(51) |  | X | X | X |  | X (waitlist control arm only) | X |
| Healthcare Usage Survey |  |  |  | X |  | X | X |
| Satisfaction & Adherence Scale |  |  |  | X |  |  | X |
| Usefulness, Satisfaction, and Ease-of-Use Scale(52) |  |  |  | X |  |  | X |
| The Gamification User Types Hexad Questionnaire (53) |  | X |  |  | X |  |  |

2.7 *Qualitative inquiry*

A descriptive qualitative approach(54) will be used to explore participants’ experiences of the eMPower program. Interviews will be offered to all participants who agree at baseline to be contacted. Semi-structured interviews will be guided by the COM-B framework(30, 43) and will explore perceived impacts on anxiety and depression symptoms, barriers and facilitators to program participation, and the acceptability of specific program elements. Individual interviews will be conducted via videoconference (~60 minutes) and transcripts available for analysis by the team.

2.8 *Economic evaluation*

Basic economic appraisal information will include: (1) health related quality of life measured using the EQ-5D-5L(49), with utility values estimated using the Canadian tariff from Xie et al (55); and (2) healthcare utilization, morbidity, and mortality captured by an end-of-study survey at 12-weeks. Albertan participants who consent to sharing their Personal Healthcare Number will have their administrative or chart data available for future collection and analysis.

2.9 *Oversight and monitoring*

A data safety monitoring board was deemed unnecessary for this study. The trial steering committee is comprised of the study statistician, study PI, and three clinical team members.

3 **Statistical method**
  3.1 *Sample size estimation*

The sample size for the HADS total primary outcome was conducted using G*power (Version 3.1.9.7)(56). The individual data from our two previous RCTs carried out in single condition group populations of IBD and PBC compared an intervention similar to eMPower study arm 3 against waitlist controls with effect sizes of 0.8 and 0.5, respectively(18, 19). Assuming an effect size of 0.2 in arm 2 (eMPower) and 0.5 in arm 3 (eMPower + brief 1-to-1 weekly check-ins), with an alpha of 0.05 and 80% power, a sample of 309 participants (103 per arm) will be adequate to detect a statistically significant mean difference(57) in the HADS total primary outcome (**Table 2)**. As an exploratory analysis, a sample size calculation was also carried out to determine how many participants will be required to detect a statistically significant mean difference between the two intervention arms (arm 2 (eMPower) and arm 3 (eMPower + 1-to-1 weekly check-ins)). Assuming an effect size of 0.2 in arm 2 and 0.5 in arm 3, this will require a sample size of 864 participants (288 per arm). Within the time and funding limitations of the grant, we will enroll at least 309 participants up to a maximum of 864 participants.

**Table 2.** Sample size contingency table

| Arm 1 (waitlist controls) | Arm 2 (eMPower) | Arm 3 (eMPower + check-ins) | Comparison tested | Total Sample Size | Total Sample Size + 20% drop out |
| --- | --- | --- | --- | --- | --- |
| 0 | 0.2 | 0.4 | Omnibus | 366 | 459 |
|  |  |  | Arm 1 v Arm 2 | 1524 | 1905 |
|  |  |  | Arm 2 v Arm 3 | 1527 | 1920 |
|  |  |  | Arm 3 vs Arm 1 | 378 | 474 |
| 0 | 0.2 | 0.5 | Omnibus | 231 | 291 |
|  |  |  | Arm 1 v Arm 2 | 1524 | 1905 |
|  |  |  | Arm 2 v Arm 3 | 690 | 864 |
|  |  |  | Arm 3 vs Arm 1 | 246 | 309 |

*Adapted from Brooks and Johanson (2011)*(57)

For the qualitative data evaluation, a sample size of ~20% for each chronic health condition group is anticipated to be sufficient for maximum variation to understand program impacts. Participants will also be purposefully sampled(58) to ensure maximum diversity across sex, ethnicity, gender, and technology proficiency. Additional participants will be recruited to achieve maximum information power(59).

3.2 *Analysis*

Descriptive statistics will be presented for the clinical and demographic characteristics and outcome variables. All analyses will adhere to the intention-to-treat-principle (ITT). The primary analysis will be based on the differences in the change in the HADS total score between arm 3 (eMPower + 1-to-1 weekly check-ins) and arm 1 (waitlist control). Previous RCT data indicates similar variances in HADS scores at baseline and end-of-program, allowing for the use of ANOVA. The analyses of secondary and exploratory outcomes (e.g., HADS subscale scores, quality of life, fatigue, frailty) will be similar to the primary outcome. With sufficient power, additional analyses will explore comparisons of interest including arm 2 (eMPower) versus study arm 3 (eMPower + 1-to-1 weekly check-ins) and control vs arm 2 (eMPower). Per-protocol analyses will be explored, with results presented if substantial differences are observed compared to the intention-to-treat group. Exploratory analysis will adjust models for clinically relevant variables (e.g., age, sex, baseline technological proficiency, baseline HADS-A and HADS-D scores) to examine their potential effects on outcomes and program engagement.

To assess the sustainability of intervention effects post-program completion, repeated measures ANOVA will analyze HADS and secondary and exploratory measures (e.g., quality of life) at 12 and 20 weeks after baseline, with time as a within-subject factor and treatment as a between-subjects factor. All analyses will utilize SAS version 9.4(60).

In collaboration with the Institute of Health Economics, a cost-utility analysis will estimate the incremental cost per Quality Adjusted Life Year (QALY) gained for each study arm using the EQ-5D-5L(49) at the same timepoints as other surveys (**Table 1**) and a self-created healthcare usage survey, collected at week 12, including questions about the frequency of primary care, specialty care, emergency department, and inpatient admissions, and whether visits were virtual or in-person. For participants in Alberta who consent to providing their personal health care number, this data can be verified using administrative health data records. Results will be presented in the Net Benefit framework, considering data from the EQ-5D-5L and resource use (e.g., self-reported mental health visits) to evaluate costs from the perspective of a publicly funded provincial payer.

3.3 *Qualitative analysis*

A reflexive thematic analysis(61) approach will be employed. An inductive approach will be used to generate initial codes, which will then be organized into categories and larger themes. Data generation and analysis will be concurrent and iterative to refine interview questions and assess information power(59). Quirkos will be used to manage the data(62).

4 **Patient and public involvement**

Patient partner organizations and 2-3 adult patient partners with lived experience of the chronic health conditions included in this study (n=20) provided feedback on the initial version of the online program. They suggested changes to the layout and content and provided ideas for the ‘Tip from your care team’ topics specific to their conditions. Changes based on their feedback included adding ~10-minute options for mindful movement, breathwork, and meditation content in addition to the original ~35-minute and ~20-minute sessions. Feedback was also sought on survey length, outcome priorities, and the structure of orientations and delivery of live group movement sessions.

5 **Discussion**

Chronic health conditions significantly contribute to global morbidity and mortality(1). Individuals living with these conditions experience a heavy symptom burden, including elevated rates of depression and anxiety(3). Online mind-body wellness programs hold potential for enhancing mental and physical health in this population. Studies such as eMPower, incorporating a mixed-methods approach across various chronic health conditions, hold promise to advance our understanding of the impact of these interventions.

This study has multiple strengths, including an RCT design, partnership with patient organizations to increase relevance, a large sample size, the inclusion of diverse chronic health conditions, programming that combines psychology-based and additional mind-body activities, automated activity tracking, and the inclusion of COM-B surveys to evaluate behavioral drivers of program engagement and behaviour change. The flexible online platform allows for the delivery of tailored elements to each chronic condition group, while retaining uniformity across remaining (90%) program content. The fully virtual nature of the program from recruitment to follow-up reduces geographic limitations for participation allowing for international study recruitment. The findings will add to a growing experience with online mind-body wellness interventions(17) in a world where an increasing number of individuals have access to internet-connected devices(14, 63).

We acknowledge several study limitations. First, unlike our previous RCTs(18, 19) which included single chronic condition groups, eMPower includes multiple chronic health conditions. This expansion was purposeful to assess the benefits of the programming across multiple, diverse groups, but its impact on the effect size of the intervention is uncertain. Second, the waitlist control group receives weekly motivational emails, a control condition that we have used in our previous research(18, 19), that is above standard care and has the potential to reduce our anticipated effect size(64, 65). Third, we did not set a minimum HADS score threshold for inclusion. This decision was agreed upon after consultation with our patient partner organizations who prioritized the inclusion of all consenting participants who reached out for support. The mitigation of these three limitations will be achieved by over-recruitment beyond the primary outcome sample size to what is possible within the funding timeline. Increasing the sample size will also allow for additional power for our secondary and exploratory analyses.

Allocation concealment was done via REDCap randomization and blinding of the study team (apart from the statistician) to the randomization tables. Lastly, while participants will be informed that at least 90 minutes of the Energize component has been associated with significant impact on anxiety, depression, and fatigue in our previous research, recognizing the competing priorities that individuals with chronic conditions may face in their day-to-day lives, no adherence requirement has been mandated as part of the inclusion criteria. An exploratory dose-response analysis will be carried out to assess the impact of engagement on effectiveness. Furthermore, while multiple topics are covered within the eMPower program, it is likely that future versions with an emphasis on specific symptoms (i.e., fatigue, sleep disturbance, frailty), may have a greater effect on these outcomes.

In summary, this protocol outlines a three-armed RCT designed to assess the effectiveness of an accessible 12-week online mind-body wellness program for adults managing various chronic health challenges. The findings of the eMPOwer trial are anticipated to contribute valuable insights into participant engagement and the overall impact of online mind-body interventions across a range of conditions.

**Appendix 1. SPIRIT Checklist**

SPIRIT 2013 Checklist: Recommended items to address in a clinical trial protocol and related documents*

| Section/item | Item No | Description | Addressed on page number |
| --- | --- | --- | --- |
| **Administrative information** | | |  |
| Title | 1 | Descriptive title identifying the study design, population, interventions, and, if applicable, trial acronym | ______1_______ |
| Trial registration | 2a | Trial identifier and registry name. If not yet registered, name of intended registry | ________3_____ |
|  | 2b | All items from the World Health Organization Trial Registration Data Set | ________n/a_____ |
| Protocol version | 3 | Date and version identifier | ___________n/a__ |
| Funding | 4 | Sources and types of financial, material, and other support | _______3______ |
| Roles and responsibilities | 5a | Names, affiliations, and roles of protocol contributors | _______1,2______ |
|  | 5b | Name and contact information for the trial sponsor | __________n/a___ |
|  | 5c | Role of study sponsor and funders, if any, in study design; collection, management, analysis, and interpretation of data; writing of the report; and the decision to submit the report for publication, including whether they will have ultimate authority over any of these activities | _____3________ |
|  | 5d | Composition, roles, and responsibilities of the coordinating centre, steering committee, endpoint adjudication committee, data management team, and other individuals or groups overseeing the trial, if applicable (see Item 21a for data monitoring committee) | _________n/a____ |
| Introduction |  |  |  |
| Background and rationale | 6a | Description of research question and justification for undertaking the trial, including summary of relevant studies (published and unpublished) examining benefits and harms for each intervention | ____6, 7________ |
|  | 6b | Explanation for choice of comparators | ______6_______ |
| Objectives | 7 | Specific objectives or hypotheses | ______7_______ |
| Trial design | 8 | Description of trial design including type of trial (eg, parallel group, crossover, factorial, single group), allocation ratio, and framework (eg, superiority, equivalence, noninferiority, exploratory) | _________7____ |
| Methods: Participants, interventions, and outcomes | | |  |
| Study setting | 9 | Description of study settings (eg, community clinic, academic hospital) and list of countries where data will be collected. Reference to where list of study sites can be obtained | _______9______ |
| Eligibility criteria | 10 | Inclusion and exclusion criteria for participants. If applicable, eligibility criteria for study centres and individuals who will perform the interventions (eg, surgeons, psychotherapists) | _______9______ |
| Interventions | 11a | Interventions for each group with sufficient detail to allow replication, including how and when they will be administered | ______10-13_ |
|  | 11b | Criteria for discontinuing or modifying allocated interventions for a given trial participant (eg, drug dose change in response to harms, participant request, or improving/worsening disease) | ____n/a_________ |
|  | 11c | Strategies to improve adherence to intervention protocols, and any procedures for monitoring adherence (eg, drug tablet return, laboratory tests) | _____14________ |
|  | 11d | Relevant concomitant care and interventions that are permitted or prohibited during the trial | ________9_____ |
| Outcomes | 12 | Primary, secondary, and other outcomes, including the specific measurement variable (eg, systolic blood pressure), analysis metric (eg, change from baseline, final value, time to event), method of aggregation (eg, median, proportion), and time point for each outcome. Explanation of the clinical relevance of chosen efficacy and harm outcomes is strongly recommended | __14-17________ |
| Participant timeline | 13 | Time schedule of enrolment, interventions (including any run-ins and washouts), assessments, and visits for participants. A schematic diagram is highly recommended (see Figure) | ______12___ |
| Sample size | 14 | Estimated number of participants needed to achieve study objectives and how it was determined, including clinical and statistical assumptions supporting any sample size calculations | ________18_____ |
| Recruitment | 15 | Strategies for achieving adequate participant enrolment to reach target sample size | _______9______ |
| **Methods: Assignment of interventions (for controlled trials)** | | |  |
| Allocation: |  |  |  |
| Sequence generation | 16a | Method of generating the allocation sequence (eg, computer-generated random numbers), and list of any factors for stratification. To reduce predictability of a random sequence, details of any planned restriction (eg, blocking) should be provided in a separate document that is unavailable to those who enrol participants or assign interventions | _____10________ |
| Allocation concealment mechanism | 16b | Mechanism of implementing the allocation sequence (eg, central telephone; sequentially numbered, opaque, sealed envelopes), describing any steps to conceal the sequence until interventions are assigned | ___10__________ |
| Implementation | 16c | Who will generate the allocation sequence, who will enrol participants, and who will assign participants to interventions | ____10_________ |
| Blinding (masking) | 17a | Who will be blinded after assignment to interventions (eg, trial participants, care providers, outcome assessors, data analysts), and how | ______10_______ |
|  | 17b | If blinded, circumstances under which unblinding is permissible, and procedure for revealing a participant’s allocated intervention during the trial | _______n/a______ |
| **Methods: Data collection, management, and analysis** | | |  |
| Data collection methods | 18a | Plans for assessment and collection of outcome, baseline, and other trial data, including any related processes to promote data quality (eg, duplicate measurements, training of assessors) and a description of study instruments (eg, questionnaires, laboratory tests) along with their reliability and validity, if known. Reference to where data collection forms can be found, if not in the protocol | __13-17_______ |
|  | 18b | Plans to promote participant retention and complete follow-up, including list of any outcome data to be collected for participants who discontinue or deviate from intervention protocols | _____16________ |
| Data management | 19 | Plans for data entry, coding, security, and storage, including any related processes to promote data quality (eg, double data entry; range checks for data values). Reference to where details of data management procedures can be found, if not in the protocol | ___15__________ |
| Statistical methods | 20a | Statistical methods for analysing primary and secondary outcomes. Reference to where other details of the statistical analysis plan can be found, if not in the protocol | __19-21________ |
|  | 20b | Methods for any additional analyses (eg, subgroup and adjusted analyses) | _20____________ |
|  | 20c | Definition of analysis population relating to protocol non-adherence (eg, as randomised analysis), and any statistical methods to handle missing data (eg, multiple imputation) | ______20_______ |
| **Methods: Monitoring** | | |  |
| Data monitoring | 21a | Composition of data monitoring committee (DMC); summary of its role and reporting structure; statement of whether it is independent from the sponsor and competing interests; and reference to where further details about its charter can be found, if not in the protocol. Alternatively, an explanation of why a DMC is not needed | __17-18_______ |
|  | 21b | Description of any interim analyses and stopping guidelines, including who will have access to these interim results and make the final decision to terminate the trial | ___n/a__________ |
| Harms | 22 | Plans for collecting, assessing, reporting, and managing solicited and spontaneously reported adverse events and other unintended effects of trial interventions or trial conduct | ______n/a_______ |
| Auditing | 23 | Frequency and procedures for auditing trial conduct, if any, and whether the process will be independent from investigators and the sponsor | _______n/a______ |
| Ethics and dissemination | | |  |
| Research ethics approval | 24 | Plans for seeking research ethics committee/institutional review board (REC/IRB) approval | _____9________ |
| Protocol amendments | 25 | Plans for communicating important protocol modifications (eg, changes to eligibility criteria, outcomes, analyses) to relevant parties (eg, investigators, REC/IRBs, trial participants, trial registries, journals, regulators) | ______n/a_______ |
| Consent or assent | 26a | Who will obtain informed consent or assent from potential trial participants or authorised surrogates, and how (see Item 32) | _______9______ |
|  | 26b | Additional consent provisions for collection and use of participant data and biological specimens in ancillary studies, if applicable | _____n/a________ |
| Confidentiality | 27 | How personal information about potential and enrolled participants will be collected, shared, and maintained in order to protect confidentiality before, during, and after the trial | __________9___ |
| Declaration of interests | 28 | Financial and other competing interests for principal investigators for the overall trial and each study site | _____2-3__ |
| Access to data | 29 | Statement of who will have access to the final trial dataset, and disclosure of contractual agreements that limit such access for investigators | _______19______ |
| Ancillary and post-trial care | 30 | Provisions, if any, for ancillary and post-trial care, and for compensation to those who suffer harm from trial participation | _______n/a______ |
| Dissemination policy | 31a | Plans for investigators and sponsor to communicate trial results to participants, healthcare professionals, the public, and other relevant groups (eg, via publication, reporting in results databases, or other data sharing arrangements), including any publication restrictions | ______21_______ |
|  | 31b | Authorship eligibility guidelines and any intended use of professional writers | _______n/a______ |
|  | 31c | Plans, if any, for granting public access to the full protocol, participant-level dataset, and statistical code | ____n/a_________ |
| Appendices |  |  |  |
| Informed consent materials | 32 | Model consent form and other related documentation given to participants and authorised surrogates | ________n/a_____ |
| Biological specimens | 33 | Plans for collection, laboratory evaluation, and storage of biological specimens for genetic or molecular analysis in the current trial and for future use in ancillary studies, if applicable | _______n/a______ |

*It is strongly recommended that this checklist be read in conjunction with the SPIRIT 2013 Explanation & Elaboration for important clarification on the items. Amendments to the protocol should be tracked and dated. The SPIRIT checklist is copyrighted by the SPIRIT Group under the Creative Commons “[Attribution-NonCommercial-NoDerivs 3.0 Unported](http://www.creativecommons.org/licenses/by-nc-nd/3.0/)” license.

**Appendix 2 – Meditation Topics**

Scarcity to Abundance – long and short versions

Meditation Warm-up

Morning Meditation

Smile Meditation

Breath

Daily Burst

Body Scan

5-Minute Recharge for the Busy Mind

Happiness Meditation

Flow State unguided music audio

Centering in Happiness

Walk in Nature

Progressive Muscle Relaxation – long and short versions

**Appendix 3 – Life Skills – Coping Skills Program Topics**

Week 1 – Routine Building

Week 2 – Values

Week 3 – SMART Goals

Week 4 – Rumble Strips

Week 5 – Pacing

Week 6 – Managing Thoughts

Week 7 – Managing Feelings

Week 8 – Self Compassion

Week 9 – Sleep

Week 10 – Building a Village

Week 11 – Gratitude

Week 12 – Keep it Going

**Appendix 4 – Life skills – Tip from your care team topics**

Digestive Disease

- Science behind the eMPower program
- Microbiome
- Elevator pitch
- Eating well on a budget
- Chronic Disease Distress
- Diet and inflammation
- What is protein?
- Shared decision making
- Healthy bowel movements
- What tests or procedures might I encounter in my journey with a digestive disease?
- Communicating with healthcare providers
- Information about the Canadian Digestive Health Foundation

Cirrhosis

- Science behind the eMPower program
- Microbiome
- Elevator pitch
- What is protein?
- Common symptoms in liver disease (part 1)
- Common symptoms in liver disease (part 2)
- Chronic Disease Distress
- Shared Decision Making
- What liver tests might my doctor send me for?
- My journey with cirrhosis
- CirrhosisCare.ca Resources
- Information about the Canadian Liver Foundation

PBC

- Conversation with a specialist (part 1)
- Conversation with a specialist (part 2)
- Alcohol and Cannabis
- Elevator Pitch
- What is protein?
- Microbiome
- Shared Decision Making
- Diet and inflammation
- Lab values in PBC
- Science behind the eMPower program
- Communicating with healthcare providers
- Information about the Canadian PBC Society

CKD

- Science behind the eMPower program
- Shared decision making
- Peer support and why it’s important (part 1)
- Peer support and why it’s important (part 2)
- Elevator pitch
- Lab values in CKD
- Chronic disease distress
- My experience as a patient with CKD
- Communicating with your healthcare team
- Top things to know about hemodialysis
- Common symptoms in CKD
- Information about the Canadian Kidney Foundation

Heart Failure

- 4 medications for heart failure
- Devices in heart failure (part 1)
- Devices in heart failure (part 2)
- What is protein?
- Elevator pitch
- Microbiome
- Lab values in heart failure
- Science behind the eMPower program
- Stages of heart failure
- Shared decision making
- New research in heart failure
- Information about HeartLife

Women who have experienced a cardiac event

- Heart attack symptoms reported by women
- Science behind the eMPower program
- Signs, symptoms, and language
- What is protein?
- Elevator pitch
- Microbiome
- Chronic disease distress
- Diet and inflammation
- Shared decision making
- Cardiovascular risk factors most often reported by women
- Communicating with your healthcare team
- Physiological symptoms and differences between men and women
- The importance of cardiac rehab for women

Post Transplant

- Science behind the eMPower program
- What are some common medications post-transplant?
- What is protein?
- Coping with isolation and loneliness post-transplant?
- Elevator pitch
- What is the microbiome?
- What is CMV infection? What else should I know from an infectious disease doctor?
- Shared decision making
- How to maintain a healthy weight post-transplant while also getting enough protein?
- Metabolic syndrome
- Chronic disease distress
- Information about the Canadian Donation and Transplantation Research Program

Inflammatory Bowel Disease

- Science behind the eMPower program
- Microbiome
- Diet
- Medications you may encounter in IBD (part 1)
- Medications you may encounter in IBD (part 2)
- Screening and endoscopy
- Preventative health measures in IBD (part 1)
- Preventative health measures in IBD (part 2)
- What tests or procedures might I encounter in my journey with IBD
- Information from a IBD-dietitian
- Communicating with your healthcare team
- Information about the Canadian Digestive Health Foundation (CDHF)

Other Chronic Health Conditions

- Science behind the eMPower program
- What is protein?
- Inflammation and diet
- Eating well on a budget
- Healthy bowel movements
- Chronic disease distress
- Elevator pitch
- Communicating with your healthcare team
- Microbiome
- Alcohol and cannabis
- Chronic pain
- Information about the Alberta Strategy for Patient Oriented Research – How do I get involved in research?

**References**

1.	World Health Organization. Global Health Estimates: Top 10 causes of death. 2022.

2.	Roser M, Ritchie, H. Burden of disease. Published online at OurWorldInDataorg. 2021.

3.	Canada PHAo. Aging and Chronic Diseases: A Profile of Canadian Seniors. Health Promot Chronic Dis Prev Can. 2021;41(1):30.

4.	Dossett ML, Fricchione GL, Benson H. A New Era for Mind-Body Medicine. N Engl J Med. 2020;382(15):1390-1.

5.	Hilfiker R, Meichtry A, Eicher M, Nilsson Balfe L, Knols RH, Verra ML, et al. Exercise and other non-pharmaceutical interventions for cancer-related fatigue in patients during or after cancer treatment: a systematic review incorporating an indirect-comparisons meta-analysis. Br J Sports Med. 2018;52(10):651-8.

6.	Weber M, Schnorr T, Morat M, Morat T, Donath L. Effects of Mind-Body Interventions Involving Meditative Movements on Quality of Life, Depressive Symptoms, Fear of Falling and Sleep Quality in Older Adults: A Systematic Review with Meta-Analysis. Int J Environ Res Public Health. 2020;17(18).

7.	NCI Dictionary of Cancer Terms. Mind-body practice. 2022.

8.	Borza L. Cognitive-behavioral therapy for generalized anxiety. Dialogues Clin Neurosci. 2017;19(2):203-8.

9.	Lee RT, Moore JA. Integrative Oncology and Ethics. Ethical Challenges in Oncology: Elsevier; 2017. p. 101-19.

10.	Lee SH, Cho SJ. Cognitive Behavioral Therapy and Mindfulness-Based Cognitive Therapy for Depressive Disorders. Adv Exp Med Biol. 2021;1305:295-310.

11.	Lee TY, Hsieh TC, Sung HC, Chen WL. Internet-Delivered Cognitive Behavior Therapy for Young Taiwanese Female Nursing Students with Irritable Bowel Syndrome-A Cluster Randomized Controlled Trial. Int J Environ Res Public Health. 2019;16(5).

12.	van Agteren J, Iasiello M, Lo L, Bartholomaeus J, Kopsaftis Z, Carey M, et al. A systematic review and meta-analysis of psychological interventions to improve mental wellbeing. Nature Human Behaviour. 2021;5(5):631-52.

13.	Der-Martirosian C, Shin M, Upham ML, Douglas JH, Zeliadt SB, Taylor SL. Telehealth Complementary and Integrative Health Therapies During COVID-19 at the U.S. Department of Veterans Affairs. Telemed J E Health. 2022.

14.	Wosik J, Fudim M, Cameron B, Gellad ZF, Cho A, Phinney D, et al. Telehealth transformation: COVID-19 and the rise of virtual care. J Am Med Inform Assoc. 2020;27(6):957-62.

15.	White V, Linardon J, Stone JE, Holmes-Truscott E, Olive L, Mikocka-Walus A, et al. Online psychological interventions to reduce symptoms of depression, anxiety, and general distress in those with chronic health conditions: a systematic review and meta-analysis of randomized controlled trials. Psychol Med. 2022;52(3):548-73.

16.	Baumel A, Torous J, Edan S, Kane JM. There is a non-evidence-based app for that: A systematic review and mixed methods analysis of depression- and anxiety-related apps that incorporate unrecognized techniques. J Affect Disord. 2020;273:410-21.

17.	Johnson E, Corrick S, Isley S, Vandermeer B, Dolgoy N, Bates J, et al. Mind-body internet and mobile-based interventions for depression and anxiety in adults with chronic physical conditions: A systematic review of RCTs. PLOS Digit Health. 2024;3(1):e0000435.

18.	Peerani F, Watt M, Ismond KP, Whitlock R, Ambrosio L, Hotte N, et al. A randomized controlled trial of a multicomponent online stress reduction intervention in inflammatory bowel disease. Therap Adv Gastroenterol. 2022;15:17562848221127238.

19.	Watt M, Hyde A, Johnson E, Wright GM, Vander Well S, Sadasivan C, et al. An online mind-body program improves mental health and quality of life in primary biliary cholangitis: A randomized controlled trial. Hepatology Communications. 2023;7(11):e0316.

20.	Whitley GA, Munro S, Hemingway P, Law GR, Siriwardena AN, Cooke D, et al. Mixed methods in pre-hospital research: understanding complex clinical problems. Br Paramed J. 2020;5(3):44-51.

21.	Baumel A, Muench F, Edan S, Kane JM. Objective User Engagement With Mental Health Apps: Systematic Search and Panel-Based Usage Analysis. J Med Internet Res. 2019;21(9):e14567.

22.	Cheng VWS. Recommendations for Implementing Gamification for Mental Health and Wellbeing. Frontiers in Psychology. 2020;11.

23.	Koivisto J, Malik A. Gamification for Older Adults: A Systematic Literature Review. The Gerontologist. 2021;61(7):e360-e72.

24.	Johnson D, Deterding S, Kuhn K-A, Staneva A, Stoyanov S, Hides L. Gamification for health and wellbeing: A systematic review of the literature. Internet Interv. 2016;6:89-106.

25.	Juszczak E, Altman DG, Hopewell S, Schulz K. Reporting of Multi-Arm Parallel-Group Randomized Trials: Extension of the CONSORT 2010 Statement. JAMA. 2019;321(16):1610-20.

26.	Chan AW, Tetzlaff JM, Gøtzsche PC, Altman DG, Mann H, Berlin JA, et al. SPIRIT 2013 explanation and elaboration: guidance for protocols of clinical trials. Bmj. 2013;346:e7586.

27.	Kathryn S, Lynsay M, Sharon Anne S, Peter C, Janis B, Jane MB, et al. A new framework for developing and evaluating complex interventions: update of Medical Research Council guidance. BMJ. 2021;374:n2061.

28.	Harris P, Taylor R, Minor B, Ellior V, Fernandez M, O’Neal L, et al. The REDCap consortium: Building an international comunity of software partners. J Biomed. 2019.

29.	Watt M, Spence JC, Tandon P. Development of a Theoretically Informed Web-Based Mind-Body Wellness Intervention for Patients With Primary Biliary Cholangitis: Formative Study. JMIR Form Res. 2021;5(10):e29064.

30.	Michie S, van Stralen MM, West R. The behaviour change wheel: A new method for characterising and designing behaviour change interventions. Implementation Science. 2011;6(1):42.

31.	Regulation (EU) 2016/679 of the European Parliament and of the Council of 27 April 2016 on the protection of natural persons with regard to the processing of personal data and on the free movement of such data, and repealing Directive 95/46/EC (General Data Protection Regulation) [2016] OJ L 119/1

32.	Peerani F, Watt M, Ismond KP, Whitlock R, Ambrosio L, Hotte N, et al. A randomized controlled trial of a multicomponent online stress reduction intervention in inflammatory bowel disease. Therapeutic Advances in Gastroenterology. 2022;15:17562848221127238.

33.	Hayes SC, Strosahl KD, Bunting K, Twohig M, Wilson KG. What Is Acceptance and Commitment Therapy? : Springer US; 2004. p. 3-29.

34.	Zigmond AS, Snaith RP. The hospital anxiety and depression scale. Acta Psychiatr Scand. 1983;67(6):361-70.

35.	Larson RD. Psychometric properties of the modified fatigue impact scale. Int J MS Care. 2013;15(1):15-20.

36.	Turner-Bowker D, Hogue SJ. Short Form 12 Health Survey (SF-12). Springer Netherlands; 2014. p. 5954-7.

37.	Hays RD, Kallich J, Mapes D, Coons S, Amin N, Carter WB, et al. Kidney Disease Quality of Life Short Form (KDQOL-SF &trade;), Version 1.3: A Manual for Use and Scoring. Santa Monica, CA: RAND Corporation; 1997.

38.	Younossi ZM, Guyatt G, Kiwi M, Boparai N, King D. Development of a disease specific questionnaire to measure health related quality of life in patients with chronic liver disease. Gut. 1999;45(2):295.

39.	Spertus JA, Jones PG. Development and Validation of a Short Version of the Kansas City Cardiomyopathy Questionnaire. Circulation: Cardiovascular Quality and Outcomes. 2015;8(5):469-76.

40.	Spertus JA, Winder JA, Dewhurst TA, Deyo RA, Prodzinski J, McDonell M, et al. Development and evaluation of the Seattle Angina Questionnaire: a new functional status measure for coronary artery disease. J Am Coll Cardiol. 1995;25(2):333-41.

41.	Irvine EJ, Zhou Q, Thompson AK. The Short Inflammatory Bowel Disease Questionnaire: a quality of life instrument for community physicians managing inflammatory bowel disease. CCRPT Investigators. Canadian Crohn's Relapse Prevention Trial. Am J Gastroenterol. 1996;91(8):1571-8.

42.	Ware JE, Jr., Sherbourne CD. The MOS 36-item short-form health survey (SF-36). I. Conceptual framework and item selection. Med Care. 1992;30(6):473-83.

43.	West R, Michie S. A brief introduction to the COM-B Model of behaviour and the PRIME Theory of motivation. Qeios. 2020.

44.	Aaronson NK, Ahmedzai S, Bergman B, Bullinger M, Cull A, Duez NJ, et al. The European Organization for Research and Treatment of Cancer QLQ-C30: a quality-of-life instrument for use in international clinical trials in oncology. JNCI: Journal of the National Cancer Institute. 1993;85(5):365-76.

45.	Bahat G, Ilhan B, Erdogan T, Catikkas NM, Karan MA, Drey M, et al. Simpler modified fried frailty scale as a practical tool to evaluate physical frailty: Methodological report for its cross-cultural adaptation and validation. Experimental Gerontology. 2022;166:111887.

46.	Rolfson DB, Majumdar SR, Tsuyuki RT, Tahir A, Rockwood K. Validity and reliability of the Edmonton Frail Scale. Age Ageing. 2006;35(5):526-9.

47.	Buysse DJ, Yu L, Moul DE, Germain A, Stover A, Dodds NE, et al. Development and validation of patient-reported outcome measures for sleep disturbance and sleep-related impairments. Sleep. 2010;33(6):781-92.

48.	Robinson S, Kissane DW, Brooker J, Hempton C, Michael N, Fischer J, et al. Refinement and revalidation of the demoralization scale: The DS-II-external validity. Cancer. 2016;122(14):2260-7.

49.	Group TE. EuroQol-a new facility for the measurement of health-related quality of life. Health policy. 1990;16(3):199-208.

50.	Tandon P, Purdy G, Ismond KP, Cruz C, Etruw E, Suderman K, et al. Heal-me PiONEer (personalized online nutrition and exercise): An RCT assessing 2 levels of app-based programming in individuals with chronic disease. Contemp Clin Trials. 2022;118:106791.

51.	Best R, Strough J, Bruine de Bruin W. Age differences in psychological distress during the COVID-19 pandemic: March 2020 - June 2021. Front Psychol. 2023;14:1101353.

52.	Gao M, Kortum P, Oswald F. Psychometric Evaluation of the USE (Usefulness, Satisfaction, and Ease of use) Questionnaire for Reliability and Validity. Proceedings of the Human Factors and Ergonomics Society Annual Meeting. 2018;62(1):1414-8.

53.	Tondello GF, Wehbe RR, Diamond L, Busch M, Marczewski A, Nacke LE. The Gamification User Types Hexad Scale. Proceedings of the 2016 Annual Symposium on Computer-Human Interaction in Play; Texas, USA. Association for Computing Machinery; 2016. p. 229–43.

54.	Bradshaw C, Atkinson S, Doody O. Employing a Qualitative Description Approach in Health Care Research. Glob Qual Nurs Res. 2017;4:2333393617742282.

55.	Xie F, Pullenayegum E, Gaebel K, Bansback N, Bryan S, Ohinmaa A, et al. A Time Trade-off-derived Value Set of the EQ-5D-5L for Canada. Med Care. 2016;54(1):98-105.

56.	Faul F, Erdfelder E, Lang A-G, Buchner A. G*Power 3: A flexible statistical power analysis program for the social, behavioral, and biomedical sciences. Behavior Research Methods. 2007;39(2):175-91.

57.	Brooks G, Johanson G. Sample Size Considerations for Multiple Comparison Procedures in ANOVA. Journal of Modern Applied Statistical Methods. 2011;10:97-100.

58.	Patton MQ. Qualitative research & evaluation methods: Integrating theory and practice: Sage publications; 2014.

59.	Saunders B, Sim J, Kingstone T, Baker S, Waterfield J, Bartlam B, et al. Saturation in qualitative research: exploring its conceptualization and operationalization. Qual Quant. 2018;52(4):1893-907.

60.	SAS Institute Inc. SHaD, Cary, NC: SAS Institute Inc., 2002-2004.

61.	Clarke V, Braun V. Thematic analysis: a practical guide. Thematic Analysis. 2021:1-100.

62.	<https://www.quirkos.com> QCSRf.

63.	Curtis ME, Clingan SE, Guo H, Zhu Y, Mooney LJ, Hser YI. Disparities in digital access among American rural and urban households and implications for telemedicine-based services. J Rural Health. 2022;38(3):512-8.

64.	Yorganci E, Evans CJ, Johnson H, Barclay S, Murtagh FE, Yi D, et al. Understanding usual care in randomised controlled trials of complex interventions: A multi-method approach. Palliat Med. 2020;34(5):667-79.

65.	Au J, Gibson BC, Bunarjo K, Buschkuehl M, Jaeggi SM. Quantifying the Difference between Active and Passive Control Groups in Cognitive Interventions Using two Meta-Analytical Approaches. J Cogn Enhanc. 2020;4(2):192-210.
