# Supplementary material for: Effect of a digital intervention on mental health symptoms in adults with chronic conditions: A three-arm randomized controlled trial
Source: PLoS Med. 2026 Aug 20;23(8):e1005198. doi: 10.1371/journal.pmed.1005198 (PMC13492819; doi:10.1371/journal.pmed.1005198)
Supplement: Supplementary file 14 [file pmed.1005198.s013.docx]

| Digestive disease | - Science behind the eMPower program - Microbiome - Elevator pitch - Eating well on a budget - Chronic Disease Distress - Diet and inflammation - What is protein? - Shared decision making - Healthy bowel movements - What tests or procedures might I encounter in my journey with a digestive disease? - Communicating with healthcare providers - Information about the Canadian Digestive Health Foundation |
| --- | --- |
| Cirrhosis | - Science behind the eMPower program - Microbiome - Elevator pitch - What is protein? - Common symptoms in liver disease (part 1) - Common symptoms in liver disease (part 2) - Chronic Disease Distress - Shared Decision Making - What liver tests might my doctor send me for? - My journey with cirrhosis - CirrhosisCare.ca Resources - Information about the Canadian Liver Foundation |
| PBC | - Conversation with a specialist (part 1) - Conversation with a specialist (part 2) - Alcohol and Cannabis - Elevator Pitch - What is protein? - Microbiome - Shared Decision Making - Diet and inflammation - Lab values in PBC - Science behind the eMPower program - Communicating with healthcare providers - Information about the Canadian PBC Society |
| PSC | - Science behind breathwork and meditation - Nutrition in PSC - What is PSC, what causes PSC, and what role do genetics play in PSC? Part 1 - What is PSC, what causes PSC, and what role do genetics play in PSC? Part 2 - Elevator pitch/coping with anger - IBD and PSC. Part I - IBD and PSC. Part II - Convo with a specialist –Q&A - Communicating with your healthcare team - What is protein and why is it so important? - PSC and liver transplantation - What is PSC Partners Seeking a Cure - Canada and USA - Registry and Faces of PSC |
| CKD | - Science behind the eMPower program - Shared decision making - Peer support and why it’s important (part 1) - Peer support and why it’s important (part 2) - Elevator pitch - Lab values in CKD - Chronic disease distress - My experience as a patient with CKD - Communicating with your healthcare team - Top things to know about hemodialysis - Common symptoms in CKD - Information about the Canadian Kidney Foundation |
| Heart failure | - 4 medications for heart failure - Devices in heart failure (part 1) - Devices in heart failure (part 2) - What is protein? - Elevator pitch - Microbiome - Lab values in heart failure - Science behind the eMPower program - Stages of heart failure - Shared decision making - New research in heart failure - Information about HeartLife |
| Post-transplant | - Science behind the eMPower program - What are some common medications post-transplant? - What is protein? - Coping with isolation and loneliness post-transplant? - Elevator pitch - What is the microbiome? - What is CMV infection? What else should I know from an infectious disease doctor? - Shared decision making - How to maintain a healthy weight post-transplant while also getting enough protein? - Metabolic syndrome - Chronic disease distress - Information about the Canadian Donation and Transplantation Research Program |
| “Other” chronic health condition | - Science behind the eMPower program - What is protein? - Inflammation and diet - Eating well on a budget - Healthy bowel movements - Chronic disease distress - Elevator pitch - Communicating with your healthcare team - Microbiome - Alcohol and cannabis - Chronic pain - Information about the Alberta Strategy for Patient Oriented Research – How do I get involved in research? |
